# Supplementary material for: Impacts of type 2 diabetes mellitus and hypertension on the incidence of cardiovascular diseases and stroke in China real-world setting: a retrospective cohort study
Source: BMJ Open. 2021 Nov 29;11(11):e053698. doi: 10.1136/bmjopen-2021-053698 (PMC8634005; doi:10.1136/bmjopen-2021-053698)
Supplement: Author's manuscript [file bmjopen-2021-053698.draft_revisions.pdf]

BMJ Open is committed to open peer review. As part of this commitment we make the peer review history of every article we publish publicly available.

When an article is published we post the peer reviewers' comments and the authors' responses online. We also post the versions of the paper that were used during peer review. These are the versions that the peer review comments apply to.

The versions of the paper that follow are the versions that were submitted during the peer review process. They are not the versions of record or the final published versions. They should not be cited or distributed as the published version of this manuscript.

BMJ Open is an open access journal and the full, final, typeset and author-corrected version of record of the manuscript is available on our site with no access controls, subscription charges or pay-per-view fees (http://bmjopen.bmj.com).

If you have any questions on BMJ Open's open peer review process please email info.bmjopen@bmj.com

BMJ Open

Impacts of type 2 diabetes mellitus and hypertension on cardiovascular disease and stroke in Chinese patients

Journal:	*BMJ Open*
Manuscript ID	bmjopen-2021-053698
Article Type:	Original research
Date Submitted by the Author:	24-Jun-2021
Complete List of Authors:	Liu, Yan; The Third People's Hospital of Datong, Department of Endocrinology<br>Li, Jie; The Third People's Hospital of Datong, Department of Endocrinology<br>Dou, Ying; Shanghai Ashermed Medical Technology Co LTD, Department of Medicine<br>ma, hongshan; The Third People's Hospital of Datong, Department of Cardiology
Keywords:	Hypertension < CARDIOLOGY, Stroke < NEUROLOGY, DIABETES & ENDOCRINOLOGY

SCHOLARONE™
Manuscripts

I, the Submitting Author has the right to grant and does grant on behalf of all authors of the Work (as defined in the below author licence), an exclusive licence and/or a non-exclusive licence for contributions from authors who are: i) UK Crown employees; ii) where BMJ has agreed a CC-BY licence shall apply, and/or iii) in accordance with the terms applicable for US Federal Government officers or employees acting as part of their official duties; on a worldwide, perpetual, irrevocable, royalty-free basis to BMJ Publishing Group Ltd ("BMJ") its licensees and where the relevant Journal is co-owned by BMJ to the co-owners of the Journal, to publish the Work in this journal and any other BMJ products and to exploit all rights, as set out in our licence.

The Submitting Author accepts and understands that any supply made under these terms is made by BMJ to the Submitting Author unless you are acting as an employee on behalf of your employer or a postgraduate student of an affiliated institution which is paying any applicable article publishing charge ("APC") for Open Access articles. Where the Submitting Author wishes to make the Work available on an Open Access basis (and intends to pay the relevant APC), the terms of reuse of such Open Access shall be governed by a Creative Commons licence – details of these licences and which Creative Commons licence will apply to this Work are set out in our licence referred to above.

Other than as permitted in any relevant BMJ Author's Self Archiving Policies, I confirm this Work has not been accepted for publication elsewhere, is not being considered for publication elsewhere and does not duplicate material already published. I confirm all authors consent to publication of this Work and authorise the granting of this licence.

For peer review only

Impacts of type 2 diabetes mellitus and hypertension on cardiovascular disease and stroke in Chinese patients

Yan Liu[1], Jie Li[1], Ying Dou[2], Hongshan Ma[3,*]

[1]Department of Endocrinology, The Third People's Hospital of Datong, Datong 037008, China.

[2]Department of Medicine, Shanghai Ashermed Medical Technology Co., LTD, Shanghai 200030, China.

[3]Department of Cardiology, The Third People's Hospital of Datong, Datong 037008, China.

*Corresponding author

Hongshan Ma, Department of Cardiology, The Third People's Hospital of Datong, Datong 037008, China. Tel: +86-13681971726, Email: mahongshannew@163.com.

Short title: Impacts of T2DM and HTN on VE and stroke

For peer review only

For peer review only - http://bmjopen.bmj.com/site/about/guidelines.xhtml

ABSTRACT

Introduction The prevalence of type 2 diabetes mellitus (T2DM) and hypertension (HTN) was largely increased in recent years. This study was performed for investigating the association of T2DM and HTN with combined vascular event (VE) and stroke risk.

Methods Patients aged≥18 with diagnosis of T2DM and/or HTN were included from SuValue database. Non-T2DM and non-HTN patients were also included in this study as control. All patients were followed up for about 5 years.

Findings For the current study, 8,012 patients with T2DM, 9,653 patients with HTN and 3,592 patients with both T2DM, 10,561 patients without T2DM or HTN were included. T2DM was significantly associated with VE and stroke risk (hazard ratio 1.332, 95% CI 1.134-1.565 and HR 1.584, 95% CI 1.246-2.014, respectively; all P values<0.001). HTN was significantly associated with VE and stroke risk (HR 3.244, 95% CI 2.946-3.572 and HR 4.543, 95% CI 3.918-5.268, respectively; all P values <0.0001). T2DM combined with HTN was significantly associated with VE and stroke risk (HR 3.002, 95% CI 2.577-3.497 and HR 4.151, 95% CI 3.346-5.149, respectively; all P values <0.0001). HTN affected the risk of VE and stroke similarly in T2DM and non-T2DM subjects. HTN is more strongly associated with VE and stroke risk than T2DM (HR 2.435, 95% CI 2.113-2.805 and HR 2.868, 95% CI 2.341-3.513, respectively; all P values <0.0001).

Interpretation T2DM and/or HTN is strongly associated with VE and stroke risk and HTN is more strongly associated with VE and stoke risk comparing with T2DM.

For peer review only - http://bmjopen.bmj.com/site/about/guidelines.xhtml

Keywords: type 2 diabetes mellitus, hypertension, cardiovascular events, stroke, vascular events

Strengths and limitations of this study

- This study included a large number of patients through hospital data (a total of 31,818 patients.
- Data information about cardiovascular risk factors were collected in this study.
- BMI and life style such as smoking and alcohol drinking were not accessed through electronic medical records.
- Mortality data were also not accessed through the HIS system in hospitals.
- We included patients who admitted to this hospital for the first time but we are not sure if he or she was firstly diagnosed as type 2 diabetes mellitus or hypertension, which make the included patients were heterogeneity.

For peer review only

INTRODUCTION

The prevalence of type 2 diabetes mellitus (T2DM) has increased to 10.4% in 2013 from 0.67% in 1980 in China.[1] Hypertension (HTN) is found to be in over two-thirds of patients with common in diabetes patients and diabetes may causally affect HTN.[2 3] The development of HTN coincides with the development of hyperglycaemia.[2]

Diabetes or HTN alone has been reported to be strong risk factor for cardiovascular diseases, all-cause mortality, coronary heart disease, ischemic heart disease and stroke in older adults in many studies.[4-13] According to the Framingham Heart Study, the rate of cardiovascular disease in adults with diabetes has absolute 2-fold risk of cardiovascular diseases comparing with subjects without diabetes.[14] Total cardiovascular disease burden in the diabetes patients has increased throughout the past four decades.[2] A cross-sectional study including newly diagnosed diabetes patients recruited for the UK prospective diabetes study showed that HTN is commonly in newly diabetes and HTN patients had a higher prevalence of cardiovascular events than normotensive subjects before the diagnosis of diabetes.[15] The co-existence of diabetes and HTN demonstrated a dramatically increased risk of cardiovascular disease by two to four times compared to the adults without HTN or diabetes.[2] Thus, diabetes and HTN are thought to be the bad companions and blood pressure control is critical in the diabetes patients combined with HTN. Besides, the impact of HTN on cardiovascular risk comparing with diabetes was still controversial. A study demonstrated that HTN had stronger association with atherosclerotic cardiovascular disease than diabetes.[7] Another study showed that patients with controlled diabetes have a similar

For peer review only - http://bmjopen.bmj.com/site/about/guidelines.xhtml
For peer review only

cardiovascular risk to patients with HTN but without diabetes.[2]

Effect of hypertension and diabetes on cardiovascular disease have been investigated in American, Finnish, Japanese and Iranian population.[7 16-18] However, there is no large-scale study to assess the joint effect of HTN and diabetes on the cardiovascular events in China. Based on the SuValue database, we retrospectively evaluated the impact of HTN and T2DM on the cardiovascular disease and stroke in the Chinese adults in this study.

METHODS

Study Design

SuValue database is a big-data hospital information system (HIS) database in China, including >90 million patients from 161 hospitals across 18 provinces in China.[19] This is a retrospective cross-sectional study designed to evaluate the risk of cardiovascular and stroke in patients with T2DM and/or HTN from 2004 to 2015. Patients were included if they met the following criteria: (1) aged≥18; (2) first outpatient visit to hospitals (no diagnosis or medication records in previous records); (3) diagnosed with T2DM only and/or HTN; (4) had the baseline examination records before or within 3 months since diagnosis (for details see the Section Baseline parameters); (5) follow-up time larger than 1 time per year. Patients were excluded: sex information missing; diagnosed with T2DM and/or HTN but without medication records; had been diagnosed with stroke, myocardial infarction, coronary heart disease, heart failure, had received coronary artery bypass grafting or percutaneous coronary intervention; with abnormal kidney function (normal range for serum creatinine: 54-106 μmoI/L for men;

For peer review only - http://bmjopen.bmj.com/site/about/guidelines.xhtml

44-97 μmol/L for women). We also included non-T2DM and non-HTN patients who performed the baseline examination but not from obstetrics and gynecology department, cancer department, neurology department and cardiology departments. In this analysis , all patients' records were deidentified and anonymized. Authorization for SuValue database was obtained when the database was setup, so no ethics consideration or written informed patient consent was needed for this analysis.

Baseline Parameters

Sex, age, triglyceride, total cholesterol, high-density lipoprotein cholesterol (HDL-C), low-density lipoprotein cholesterol (LDL-C), serum creatinine, fasting blood glucose, serum insulin, proteinuria, medication (antidiabetics and antihypertensive medications) at baseline were captured from electronic medical records within 3 months from the diagnosis of T2DM and/or HTN or non-T2DM and non-HTN.

Outcomes and Follow-up

We examined 3 outcomes of interest, including the following: combined vascular event (VE; stroke, myocardial infarction, coronary heart disease, heart failure and coronary bypass, percutaneous coronary intervention) and stroke. The follow-up was defined by the first event of outcome or last record before December 31st 2019 in the event-free cases.

Statistical Analyses

The categorical variables were described using frequency and percentage. The continuous variables were described using mean±SD if normally distributed or median interquartile range if not. Baseline characteristics were compared for using ANOVA or

For peer review only - http://bmjopen.bmj.com/site/about/guidelines.xhtml

Chi-square tests. Cox proportional hazards model was used to assess the association between diseases and each of the outcomes, which was adjusted for cardiovascular risk factors including gender, age, triglyceride, total cholesterol, HDL-C and LDL-C. Unadjusted, sex- and age-adjusted and cardiovascular risk factors–adjusted hazard ratios were obtained. A P<0.05 (two-sided) was considered as statistically significant. All statistical analyses were performed using SAS 9.4 (SAS Institute, Cary, NC, USA).

RESULTS

For the current study, 8,012 patients with T2DM only, 9,653 patients with HTN only and 3,592 patients with both T2DM and HTN and 10,561 patients without T2DM or HTN were selected from SuValue database. The median follow-up duration was 4.1 (2.4, 4.9) years. General characteristics of the study population at baseline are presented in Table 1. Comparing baseline characteristics between the four groups revealed a significant difference except for total cholesterol and serum insulin.

Kaplan–Meier analyses demonstrated an increased combined vascular event (VE) and stroke risk according to the presence of diabetes mellitus without considering the presence of HTN (Fig. 1A). In unadjusted models, comparing with non-HTN and non-T2DM patients, prevalent diabetes mellitus was significantly associated with a 74.7% increased risk of VE [95% confidence interval (CI) 1.566-1.949, P<0.0001] and 107.7% increased risk of stroke (95% CI 1.755-2.459, P<0.0001). Further adjustment with the age and sex and then with cardiovascular risk factors attenuated the association with VE and stroke risk (HR 1.332, 95% CI 1.134-1.565 and HR 1.584, 95% CI 1.246-2.014, respectively; P<0.01, Table 2).

For peer review only - http://bmjopen.bmj.com/site/about/guidelines.xhtml

Kaplan–Meier analyses demonstrated an increased combined vascular event (VE) and stroke risk according to the presence of HTN without considering the presence of diabetes mellitus (Fig. 1B). Prevalent HTN was significantly associated with a 524.6% increased risk of VE (-95% CI 5.712-6.830) and 764.2% increased risk of stroke (95% CI 7.517-9.934) in unadjusted models (all Ps<0.0001). These associations were attenuated but still significant after adjustment for age and sex and then other major cardiovascular risk factors for both VE and stroke risk (HR 3.244, 95% CI 2.946-3.572 and HR 4.543, 95% CI 3.918-5.268, respectively; all Ps<0.0001, Table 2).

Kaplan–Meier analyses demonstrated reduced VE risk and stroke risk in those with HTN and diabetes mellitus (Fig. 1C). Prevalent HTN and T2DM was significantly associated with a 393.0% increased risk of VE (95% CI 4.93-5.474) and 499.0% increased risk of stroke (95% CI 5.102-7.032) in unadjusted models (all Ps<0.0001). These associations were attenuated but still significant after adjustment for age and sex and then other major cardiovascular risk factors for both VE risk and stroke risk (HR 3.002, 95% CI 2.577-3.497 and HR 4.151, 95% CI 3.346-5.149, respectively; all Ps<0.0001, Table 2).

Comparing with HTN, unadjusted hazard ratio for VE risk was 0.789 and for stroke risk was 0.693 in those with T2DM and HTN (all Ps<0.0001). But after adjustment with major cardiovascular risk factors, there was no significant reduced risk for T2DM and HTN comparing with HTN (Table 3).

In unadjusted model, HTN and T2DM combined with HTN were associated with the VE and stroke risk comparing with T2DM. After adjustment with age and sex and major

For peer review only - http://bmjopen.bmj.com/site/about/guidelines.xhtml

cardiovascular risk factors, prevalent HTN and T2DM combined with HTN were still significantly associated with VE and stroke risk (Table 4).

DISCUSSION

In this present study, having HTN and/or T2DM was significantly associated with VE and stroke before and after adjustment for major cardiovascular risk factors comparing with the non-T2DM and non-HTN. The association of T2DM with the risk of both VE and stoke risk attenuated after adjusting for major cardiovascular risk factors. HTN affected the risk of VE and stroke similarly in T2DM and non-T2DM patients after adjusting for major cardiovascular risk factors. Besides, HTN is more significantly associated with VE and stroke risk than T2DM.

In unadjusted analyses and adjustment model for major cardiovascular risk factors, we observed that patients with both T2DM and HTN showed increased risk of VE and stroke comparing with those with T2DM, and the hazard ratios were significantly increased from T2DM to both T2DM and HTN. However, after adjustment for major cardiovascular risk factors, patients with T2DM and HTN were not significantly increased with the VE and stroke risk comparing with HTN only. Similarly, a prospective study of 49,582 Finnish subjects showed that HTN affected that risk of stroke similarly in T2DM and non-T2DM subjects.[20] In this study, we included patients with HTN and/or T2DM having medication administration record. A meta-analysis study demonstrated that antihypertensive treatment decreased the risk of cardiovascular disease and stroke among patients with a history of CVD or diabetes but without HTN.[21] Thus, combined T2DM and HTN did not increase the risk of VE and stroke comparing

For peer review only - http://bmjopen.bmj.com/site/about/guidelines.xhtml

with HTN may be due to the antihypertensive treatment in this population.

Besides, we also observed that HTN was more associated with VE and stroke risk than T2DM alone. The results were similar with previous report that HTN was more related with all-cause and atherosclerotic cardiovascular disease than T2DM alone in community-dwelling older adults.[7] However, a study performed in Iranian older adults showed that T2DM alone increased the all-cause mortality by 62% comparing with HTN alone.[18] Thus, a prospective study was necessary to be performed for further analysis.

The current study has several significant strengths. First, this study included a large number of patients through hospital data. Second, data information about cardiovascular risk factors were collected in this study. However, there were several limitations in this study. First, BMI and life style such as smoking and alcohol drinking were not accessed through electronic medical records. Second, mortality data were also not accessed through the HIS system in hospitals. Third, we included patients who admitted to this hospital for the first time but we are not sure if he or she was firstly diagnosed as T2DM or HTN, which make the included patients were heterogeneity.

CONCLUSIONS

In summary, HTN and/or T2DM was strongly associated with the increased risk of VE and stroke independent of conventional cardiovascular risk factors. T2DM seems not the risk of VE and stroke in HTN patients after adjusting for major cardiovascular risk factors. HTN is more significantly associated with VE and stroke risk than T2DM. However, a prospective study investigating the impact of HTN and/or T2DM on VE

For peer review only - http://bmjopen.bmj.com/site/about/guidelines.xhtml

and stroke risk is needed to be performed.

For peer review only

For peer review only - http://bmjopen.bmj.com/site/about/guidelines.xhtml

ACKNOWLEDGEMENTS

None

FUNDING

The authors received no specific funding for this work.

AUTHOR CONTRIBUTIONS

HSM and YL designed the study, analyzed the data, and wrote the first draft of the manuscript. HSM, YL, JL, and YD revised it critically for important intellectual content and approved the final version.

DATA AVAILABILITY

All data relevant to the study were obtained from SuValue database which belongs to a third party and is not publicly available.

Patient and Public Involvement

None.

REFERENCES

1 Society CD. Guidelines for the prevention and treatment of type 2 diabetes in China (2017 edition). *J Chin J Diabetes* 2018;10:4-67.

2 Ferrannini E, Cushman WC. Diabetes and hypertension: the bad companions. *The Lancet* 2012;380(9841):601-10. doi: 10.1016/s0140-6736(12)60987-8

3 Sun D, Zhou T, Heianza Y, et al. Type 2 Diabetes and Hypertension. *Circ Res* 2019;124(6):930-37. doi: 10.1161/CIRCRESAHA.118.314487 [published Online First: 2019/01/17]

4 Kannel WB, Neaton JD, Wentworth D, et al. Overall and coronary heart disease mortality rates in relation to major risk factors in 325,348 men screened for the MRFIT. Multiple Risk Factor Intervention Trial. *American heart journal* 1986;112(4):825-36. doi: 10.1016/0002-8703(86)90481-3 [published Online First: 1986/10/01]

For peer review only - http://bmjopen.bmj.com/site/about/guidelines.xhtml

5 Fox CS. Cardiovascular disease risk factors, type 2 diabetes mellitus, and the Framingham Heart Study. *Trends in cardiovascular medicine* 2010;20(3):90-5. doi: 10.1016/j.tcm.2010.08.001 [published Online First: 2010/12/07]

6 Adab P, Cheng KK, Jiang CQ, et al. Age-specific relevance of usual blood pressure to vascular mortality. *Lancet (London, England)* 2003;361(9366):1391; author reply 91-2. doi: 10.1016/s0140-6736(03)13063-2 [published Online First: 2003/04/25]

7 Oh JY, Allison MA, Barrett-Connor E. Different impacts of hypertension and diabetes mellitus on all-cause and cardiovascular mortality in community-dwelling older adults: the Rancho Bernardo Study. *J Hypertens* 2017;35(1):55-62. doi: 10.1097/HJH.0000000000001145 [published Online First: 2016/12/03]

8 Ikeda A, Iso H, Yamagishi K, et al. Blood pressure and the risk of stroke, cardiovascular disease, and all-cause mortality among Japanese: the JPHC Study. *American journal of hypertension* 2009;22(3):273-80. doi: 10.1038/ajh.2008.356 [published Online First: 2009/02/21]

9 Lotfaliany M, Akbarpour S, Mozafary A, et al. Hypertension phenotypes and incident cardiovascular disease and mortality events in a decade follow-up of a Middle East cohort. *J Hypertens* 2015;33(6):1153-61. doi: 10.1097/hjh.0000000000000540 [published Online First: 2015/02/24]

10 Sarwar N, Gao P, Seshasai SR, et al. Diabetes mellitus, fasting blood glucose concentration, and risk of vascular disease: a collaborative meta-analysis of 102 prospective studies. *Lancet (London, England)* 2010;375(9733):2215-22. doi: 10.1016/s0140-6736(10)60484-9 [published Online First: 2010/07/09]

11 Huxley R, Barzi F, Woodward M. Excess risk of fatal coronary heart disease associated with diabetes in men and women: meta-analysis of 37 prospective cohort studies. *BMJ (Clinical research ed)* 2006;332(7533):73-8. doi: 10.1136/bmj.38678.389583.7C [published Online First: 2005/12/24]

12 Schramm TK, Gislason GH, Køber L, et al. Diabetes patients requiring glucose-lowering therapy and nondiabetics with a prior myocardial infarction carry the same cardiovascular risk: a population study of 3.3 million people. *Circulation*

For peer review only - http://bmjopen.bmj.com/site/about/guidelines.xhtml

2008;117(15):1945-54. doi: 10.1161/circulationaha.107.720847 [published Online First: 2008/04/02]

13 Almdal T, Scharling H, Jensen JS, et al. The independent effect of type 2 diabetes mellitus on ischemic heart disease, stroke, and death: a population-based study of 13,000 men and women with 20 years of follow-up. *Archives of internal medicine* 2004;164(13):1422-6. doi: 10.1001/archinte.164.13.1422 [published Online First: 2004/07/14]

14 Fox CS, Coady S, Sorlie PD, et al. Trends in cardiovascular complications of diabetes. *Jama* 2004;292(20):2495-9. doi: 10.1001/jama.292.20.2495 [published Online First: 2004/11/25]

15 Hypertension in Diabetes Study (HDS): I. Prevalence of hypertension in newly presenting type 2 diabetic patients and the association with risk factors for cardiovascular and diabetic complications. *J Hypertens* 1993;11(3):309-17. doi: 10.1097/00004872-199303000-00012 [published Online First: 1993/03/01]

16 Hu G, Jousilahti P, Tuomilehto J. Joint effects of history of hypertension at baseline and type 2 diabetes at baseline and during follow-up on the risk of coronary heart disease. *Eur Heart J* 2007;28(24):3059-66. doi: 10.1093/eurheartj/ehm501 [published Online First: 2007/11/06]

17 Kokubo Y, Okamura T, Watanabe M, et al. The combined impact of blood pressure category and glucose abnormality on the incidence of cardiovascular diseases in a Japanese urban cohort: the Suita Study. *Hypertens Res* 2010;33(12):1238-43. doi: 10.1038/hr.2010.174 [published Online First: 2010/10/12]

18 Zafari N, Asgari S, Lotfaliany M, et al. Impact Of Hypertension versus Diabetes on Cardiovascular and All-cause Mortality in Iranian Older Adults: Results of 14 Years of Follow-up. *Sci Rep* 2017;7(1):14220. doi: 10.1038/s41598-017-14631-2 [published Online First: 2017/10/29]

19 Wang C, Gao Y, Zhu L, et al. Treatment Patterns in Patients With Newly Diagnosed Type 2 Diabetes in China: A Retrospective, Longitudinal Database Study. *Clinical therapeutics* 2019;41(8):1440-52. doi: 10.1016/j.clinthera.2019.05.003 [published

For peer review only - http://bmjopen.bmj.com/site/about/guidelines.xhtml

Online First: 2019/06/04]

20 Hu G, Sarti C, Jousilahti P, et al. The impact of history of hypertension and type 2 diabetes at baseline on the incidence of stroke and stroke mortality. *Stroke* 2005;36(12):2538-43. doi: 10.1161/01.Str.0000190894.30964.75 [published Online First: 2005/11/12]

21 Thompson AM, Hu T, Eshelbrenner CL, et al. Antihypertensive Treatment and Secondary Prevention of Cardiovascular Disease Events Among Persons Without Hypertension: A Meta-analysis. *JAMA* 2011;305(9):913-22. doi: 10.1001/jama.2011.250 %J JAMA

For peer review only

For peer review only - http://bmjopen.bmj.com/site/about/guidelines.xhtml

Table 1 Baseline characteristics of included patients.

	HTN only	T2DM only	T2DM and HTN	Non-T2DM and non-HTN	P value
**Total**	9,653	8,012	3,592	10561	
**Sex**					
Male	4,419 (45.8%)	4,006 (50.0%)	1,634 (45.5%)	4889 (46.3%)	-
Female	5,234 (54.2%)	4,006 (50.0%)	1,958 (54.5%)	5672 (53.7%)	-
**Age**					
18-29 (n, %)	79 (0.8%)	204 (2.5%)	31 (0.9%)	1372 (13.0%)	-
30-39 (n, %)	602 (6.2%)	831 (10.4%)	139 (3.9%)	2439 (23.1%)	-
40-49 (n, %)	1,856 (19.2%)	2,323 (29.0%)	553 (15.4%)	3227 (30.6%)	-
50-59 (n, %)	1,856 (20.7%)	2,297 (28.7%)	927 (25.8%)	1954 (18.5%)	-
60-69 (n, %)	2,552 (26.4%)	1,704 (21.3%)	1,194 (33.2%)	1179 (11.2%)	-
70-79 (n, %)	1,715 (17.8%)	556 (6.9%)	573 (16.0%)	336 (3.2%)	-
≥80 (n, %)	852 (8.8%)	97 (1.2%)	175 (4.9%)	54 (0.5%)	-
**Triglyceride (mmol/L)**	1.86 (1.54)	2.35 (2.55)	2.29 (1.85)	1.71 (1.65)	<.0001
**Total cholesterol (mmol/L)**	5.31 (1.19)	5.32 (1.41)	5.29 (1.28)	5.28 (1.15)	0.3506
**HDL-C (mmol/L)**	1.41 (0.41)	1.25 (0.45)	1.26 (0.39)	1.40 (0.39)	<.0001
**LDL-C (mmol/L)**	3.17 (1.00)	3.07 (1.03)	3.02 (0.98)	3.03 (0.90)	<.0001
**Serum creatinine (μmol/L)**	81.11 (39.35)	70.57 (23.94)	84.26 (59.38)	76.78 (20.42)	<.0001
**HbA1c (%)**	5.58 (1.38)	9.41 (3.11)	8.41 (2.63)	5.73 (1.31)	<.0001
**Fasting blood-glucose (mmol/L)**	5.76 (1.28)	11.15 (5.28)	9.88 (5.14)	5.58 (1.13)	<.0001
**Serum insulin (pmol/liter)**	95.63 (60.05)	117.64 (288.36)	78.43 (57.89)	9.98 (3.21)	0.6502
**Proteinuria (positive)**	310 (3.2%)	145 (1.8%)	126 (3.5%)	143 (1.4%)	<.0001
**Diabetes complications**					
Diabetic nephropathy	-	450 (5.6%)	446 (12.4%)	-	-
Diabetic retinopathy	-	313 (3.9%)	159 (4.4%)	-	-

For peer review only - http://bmjopen.bmj.com/site/about/guidelines.xhtml

Diabetic neuropathy	-	462 (5.8%)	225 (6.3%)	-	-
Diabetic lower limb vascular disease	-	7 (0.09%)	15 (0.42%)	-	-
Diabetic foot	-	80 (1.0%)	61 (1.7%)	-	-

HTN: hypertension; T2DM: type 2 diabetes mellitus; HDL-C: high density lipoprotein cholesterol; LDL-C: low density lipoprotein cholesterol. For continuous variables, data were presented as mean (SD) and P values for four groups were calculated using ANOVA test. For categorical variables, data were presented as number (frequency) and P values for four groups were calculated using Chi-square test.

For peer review only

For peer review only - http://bmjopen.bmj.com/site/about/guidelines.xhtml

Table 2 Association of type 2 diabetes mellitus (T2DM) and/or hypertension (HTN) with combined vascular event and stroke comparing with non-T2DM and non-HTN.

	Combined vascular event (VE)		Stroke	
	HR (95% CI)	P value	HR (95% CI)	P value
**Unadjusted**				
Non-T2DM and non-HTN	1 (reference)		1 (reference)	
T2DM only	1.747 (1.566, 1.949)	<.0001	2.077 (1.755, 2.459)	<.0001
HTN only	6.246 (5.712, 6.830)	<.0001	8.642 (7.517, 9.934)	<.0001
T2DM and HTN	4.930 (4.93, 5.474)	<.0001	5.990 (5.102, 7.032)	<.0001
**Age- and sex-adjusted**				
Non-T2DM and non-HTN	1 (reference)		1 (reference)	
T2DM only	1.258 (1.127, 1.405)	<.0001	1.464 (1.235, 1.735)	<.0001
HTN only	3.344 (3.046, 3.670)	<.0001	4.409 (3.817, 5.093)	<.0001
T2DM and HTN	2.595 (2.329, 2.892)	<.0001	3.021 (2.561, 3.563)	<.0001
**Risk factors–adjusted**				
Non-T2DM and non-HTN	1 (reference)		1 (reference)	
T2DM only	1.332 (1.134, 1.565)	0.0005	1.584 (1.246, 2.014)	<.0001
HTN only	3.244 (2.946, 3.572)	<.0001	4.543 (3.918, 5.268)	<.0001
T2DM and HTN	3.002 (2.577, 3.497)	<.0001	4.151 (3.346, 5.149)	<.0001

CI, confidence interval; VE, combined vascular event; T2DM, type 2 diabetes mellitus; HR, hazard ratio; HTN, hypertension. Risk factors include age, sex, triglycerides, total cholesterol, high density lipoprotein cholesterol, low density lipoprotein cholesterol.

For peer review only

For peer review only - http://bmjopen.bmj.com/site/about/guidelines.xhtml

Table 3 Association of type 2 diabetes mellitus (T2DM) and hypertension (HTN) with combined vascular event and stroke comparing with HTN only.

	**Combined vascular event (VE)**		**Stroke**	
	HR (95% CI)	P value	HR (95% CI)	P value
**Unadjusted**				
HTN only	1 (reference)		1 (reference)	
T2DM and HTN	0.789 (0.732, 0.851)	<.0001	0.693 (0.624, 0.769)	<.0001
**Age- and sex-adjusted**				
HTN only	1 (reference)		1 (reference)	
T2DM and HTN	0.776 (0.720, 0.836)	<.0001	0.685 (0.617, 0.761)	<.0001
**Risk factors–adjusted**				
HTN only	1 (reference)		1 (reference)	
T2DM and HTN	0.925 (0.814, 1.052)	0.2352	0.914 (0.771, 1.082)	0.2959

CI, confidence interval; VE, combined vascular event; T2DM, type 2 diabetes mellitus; HR, hazard ratio; HTN, hypertension. Risk factors include age, sex, triglycerides, total cholesterol, high density lipoprotein cholesterol, low density lipoprotein cholesterol.

For peer review only

For peer review only - http://bmjopen.bmj.com/site/about/guidelines.xhtml

Table 4 Association of type 2 diabetes mellitus (T2DM) and/or hypertension (HTN) with combined vascular event and stroke comparing with T2DM only.

	Combined vascular event (VE)		Stroke	
	HR (95% CI)	P value	HR (95% CI)	P value
**Unadjusted**				
T2DM only	1 (reference)		1 (reference)	
HTN only	3.575 (3.296, 3.877)	<.0001	4.160 (3.701, 4.675)	<.0001
T2DM and HTN	2.821 (2.558, 3.112)	<.0001	2.883 (2.504, 3.321)	<.0001
**Age- and sex-adjusted**				
T2DM only	1 (reference)		1 (reference)	
HTN only	2.657 (2.447, 2.885)	<.0001	3.011 (2.676, 3.389)	<.0001
T2DM and HTN	2.062 (1.868, 2.276)	<.0001	2.063 (1.789, 2.379)	<.0001
**Risk factors–adjusted**				
T2DM only	1 (reference)		1 (reference)	
HTN only	2.435 (2.113, 2.805)	<.0001	2.868 (2.341, 3.513)	<.0001
T2DM and HTN	2.253 (1.876, 2.706)	<.0001	2.620 (2.031, 3.380)	<.0001

CI, confidence interval; VE, combined vascular event; T2DM, type 2 diabetes mellitus; HR, hazard ratio; HTN, hypertension. Risk factors include age, sex, triglycerides, total cholesterol, high density lipoprotein cholesterol, low density lipoprotein cholesterol.

For peer review only - http://bmjopen.bmj.com/site/about/guidelines.xhtml

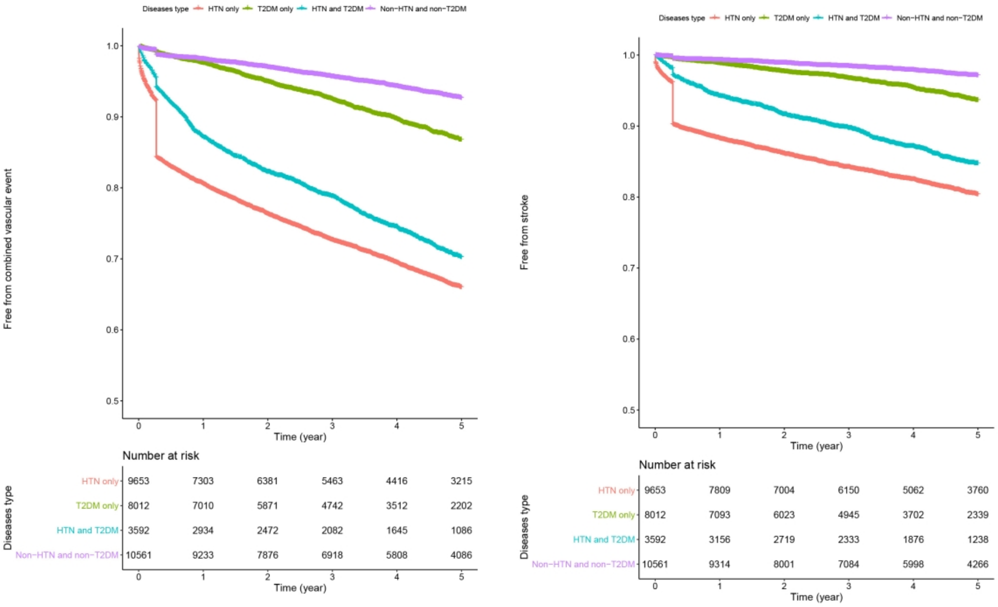

Figure 1 Kaplan–Meier survival curve of combined vascular event (VE) and stroke according to the presence of type 2 diabetes mellitus (T2DM) and hypertension (HTN).

479x294mm (150 x 150 DPI)

STROBE Statement—Checklist of items that should be included in reports of ***cross-sectional studies***

	Item No	Recommendation	Page No
**Title and abstract**	1	(*a*) Indicate the study's design with a commonly used term in the title or the abstract	1, 2
		(*b*) Provide in the abstract an informative and balanced summary of what was done and what was found	2
**Introduction**			
Background/rationale	2	Explain the scientific background and rationale for the investigation being reported	4, 5
Objectives	3	State specific objectives, including any prespecified hypotheses	5
**Methods**			
Study design	4	Present key elements of study design early in the paper	5
Setting	5	Describe the setting, locations, and relevant dates, including periods of recruitment, exposure, follow-up, and data collection	5, 6
Participants	6	(*a*) Give the eligibility criteria, and the sources and methods of selection of participants	5, 6
Variables	7	Clearly define all outcomes, exposures, predictors, potential confounders, and effect modifiers. Give diagnostic criteria, if applicable	6
Data sources/ measurement	8*	For each variable of interest, give sources of data and details of methods of assessment (measurement). Describe comparability of assessment methods if there is more than one group	*5, 6*
Bias	9	Describe any efforts to address potential sources of bias	5, 6
Study size	10	Explain how the study size was arrived at	7
Quantitative variables	11	Explain how quantitative variables were handled in the analyses. If applicable, describe which groupings were chosen and why	5, 6
Statistical methods	12	(*a*) Describe all statistical methods, including those used to control for confounding	6, 7
		(*b*) Describe any methods used to examine subgroups and interactions	NA
		(*c*) Explain how missing data were addressed	NA
		(*d*) If applicable, describe analytical methods taking account of sampling strategy	NA
		(*e*) Describe any sensitivity analyses	NA
**Results**			
Participants	13*	(a) Report numbers of individuals at each stage of study—eg numbers potentially eligible, examined for eligibility, confirmed eligible, included in the study, completing follow-up, and analysed	7
		(b) Give reasons for non-participation at each stage	NA
		(c) Consider use of a flow diagram	NA
Descriptive data	14*	(a) Give characteristics of study participants (eg demographic, clinical, social) and information on exposures and potential confounders	7, 8
		(b) Indicate number of participants with missing data for each variable of interest	7, 8
Outcome data	15*	Report numbers of outcome events or summary measures	7, 8
Main results	16	(*a*) Give unadjusted estimates and, if applicable, confounder-adjusted estimates and their precision (eg, 95% confidence interval). Make clear which confounders were adjusted for and why they were included	7, 8

For peer review only

For peer review only - http://bmjopen.bmj.com/site/about/guidelines.xhtml

		(*b*) Report category boundaries when continuous variables were categorized	7, 8
		(*c*) If relevant, consider translating estimates of relative risk into absolute risk for a meaningful time period	NA
Other analyses	17	Report other analyses done—eg analyses of subgroups and interactions, and sensitivity analyses	NA
**Discussion**			
Key results	18	Summarise key results with reference to study objectives	9, 10
Limitations	19	Discuss limitations of the study, taking into account sources of potential bias or imprecision. Discuss both direction and magnitude of any potential bias	10
Interpretation	20	Give a cautious overall interpretation of results considering objectives, limitations, multiplicity of analyses, results from similar studies, and other relevant evidence	9, 10
Generalisability	21	Discuss the generalisability (external validity) of the study results	9, 10
**Other information**			
Funding	22	Give the source of funding and the role of the funders for the present study and, if applicable, for the original study on which the present article is based	11

*Give information separately for exposed and unexposed groups.

Note: An Explanation and Elaboration article discusses each checklist item and gives methodological background and published examples of transparent reporting. The STROBE checklist is best used in conjunction with this article (freely available on the Web sites of PLoS Medicine at http://www.plosmedicine.org/, Annals of Internal Medicine at http://www.annals.org/, and Epidemiology at http://www.epidem.com/). Information on the STROBE Initiative is available at www.strobe-statement.org.

For peer review only

BMJ Open

Impacts of type 2 diabetes mellitus and hypertension on the incidence of cardiovascular diseases and stroke in China real-world setting: A retrospective, cohort study

Journal:	*BMJ Open*
Manuscript ID	bmjopen-2021-053698.R1
Article Type:	Original research
Date Submitted by the Author:	06-Oct-2021

Complete List of Authors:	Liu, Yan; The Third People's Hospital of Datong, Department of Endocrinology<br>Li, Jie; The Third People's Hospital of Datong, Department of Endocrinology<br>Dou, Ying; Shanghai Ashermed Medical Technology Co LTD, Department of Medicine<br>Ma, Hongshan; The Third People's Hospital of Datong, Department of Cardiology

**Primary Subject Heading**:	Diabetes and endocrinology
Secondary Subject Heading:	Public health
Keywords:	Hypertension < CARDIOLOGY, Stroke < NEUROLOGY, DIABETES & ENDOCRINOLOGY

SCHOLARONE™
Manuscripts

I, the Submitting Author has the right to grant and does grant on behalf of all authors of the Work (as defined in the below author licence), an exclusive licence and/or a non-exclusive licence for contributions from authors who are: i) UK Crown employees; ii) where BMJ has agreed a CC-BY licence shall apply, and/or iii) in accordance with the terms applicable for US Federal Government officers or employees acting as part of their official duties; on a worldwide, perpetual, irrevocable, royalty-free basis to BMJ Publishing Group Ltd ("BMJ") its licensees and where the relevant Journal is co-owned by BMJ to the co-owners of the Journal, to publish the Work in this journal and any other BMJ products and to exploit all rights, as set out in our licence.

The Submitting Author accepts and understands that any supply made under these terms is made by BMJ to the Submitting Author unless you are acting as an employee on behalf of your employer or a postgraduate student of an affiliated institution which is paying any applicable article publishing charge ("APC") for Open Access articles. Where the Submitting Author wishes to make the Work available on an Open Access basis (and intends to pay the relevant APC), the terms of reuse of such Open Access shall be governed by a Creative Commons licence – details of these licences and which Creative Commons licence will apply to this Work are set out in our licence referred to above.

Other than as permitted in any relevant BMJ Author's Self Archiving Policies, I confirm this Work has not been accepted for publication elsewhere, is not being considered for publication elsewhere and does not duplicate material already published. I confirm all authors consent to publication of this Work and authorise the granting of this licence.

For peer review only

Impacts of type 2 diabetes mellitus and hypertension on the incidence of cardiovascular diseases and stroke in China real-world setting: A retrospective, cohort study

Yan Liu[1], Jie Li[1], Ying Dou[2], Hongshan Ma[3,*]

[1]Department of Endocrinology, The Third People's Hospital of Datong, Datong 037008, China.

[2]Department of Medicine, Shanghai Ashermed Medical Technology Co., LTD, Shanghai 200030, China.

[3]Department of Cardiology, The Third People's Hospital of Datong, Datong 037008, China.

*Corresponding author

Hongshan Ma, Department of Cardiology, The Third People's Hospital of Datong, Datong 037008, China. Tel: +86-13681971726, Email: mahongshannew@163.com.

Short title: Impacts of T2DM and HTN on combined VE and stroke risk

For peer review only - http://bmjopen.bmj.com/site/about/guidelines.xhtml

ABSTRACT

Objective The prevalence of type 2 diabetes mellitus (T2DM) and hypertension (HTN) has largely increased in recent years. However, there is little evidence from large-scale study to assess the joint effect of T2DM and HTN on the risk of cardiovascular events in China. This study was performed to investigate the association of T2DM and HTN with the incidence of combined vascular event (VE) and stroke in China.

Design A retrospective cohort study.

Setting Data were collected from the SuValue database which includes electronic medical records of >90 million patients from 161 hospitals across 18 provinces in China.

Participants Patients aged≥18 with diagnosis of T2DM and/or HTN were included. Non-T2DM and non-HTN patients were included in this study as control.

Outcomes Incidence of combined vascular event (VE) and stroke during the study.

Results For the current study, 8,012 patients with T2DM, 9,653 patients with HTN and 3,592 patients with both T2DM and HTN, 10,561 patients without T2DM or HTN were included. T2DM was significantly associated with combined VE and stroke risk (hazard ratio 1.332, 95% CI 1.134-1.565 and HR 1.584, 95% CI 1.246-2.014, respectively). HTN was significantly associated with combined VE and stroke risk (HR 3.244, 95% CI 2.946-3.572 and HR 4.543, 95% CI 3.918-5.268, respectively). T2DM combined with HTN was significantly associated with combined VE and stroke risk (HR 3.002, 95% CI 2.577-3.497 and HR 4.151, 95% CI 3.346-5.149, respectively). HTN was more strongly associated with combined VE and stroke risk than T2DM (HR 2.435, 95% CI 2.113-2.805 and HR 2.868, 95% CI 2.341-3.513, respectively).

For peer review only - http://bmjopen.bmj.com/site/about/guidelines.xhtml

Conclusion Subjects with T2DM and HTN were strongly associated with combined VE and stroke risk, however, the HTN only group was more strongly associated with combined VE and stroke risk compared with the T2DM only group.

Keywords: type 2 diabetes mellitus, hypertension, cardiovascular events, stroke, vascular events

Strengths and limitations of this study

Strengths of this study

- This study included large number of patients in real-world setting (a total of 31,818 patients).
- Cardiovascular risk factors were collected in this study.

Limitations of this study

- BMI and life style such as smoking and alcohol drinking were not accessed through electronic medical records in hospitals.
- Mortality data were not accessed through the electronic medical records in hospitals.

For peer review only - http://bmjopen.bmj.com/site/about/guidelines.xhtml

INTRODUCTION

The prevalence of type 2 diabetes mellitus (T2DM) has increased to 10.4% in 2013 and 11.2% in 2015 from 0.67% in 1980 in China.[1 2] Hypertension (HTN) has been found to be common in over two-thirds of patients with diabetes.[3 4] The development of HTN coincides with the development of hyperglycaemia.[3] Insulin resistance and hyperinsulinaemia might promote atherogenesis, thereby affecting blood pressure homoeostasis.[5 6]

Diabetes has been reported to be strong risk factor for cardiovascular diseases, all-cause mortality, coronary heart disease, ischemic heart disease and stroke in many studies.[7-11] According to the Framingham Heart Study, adults with diabetes had absolute 2-fold risk of cardiovascular diseases compared with subjects without diabetes.[12] Total cardiovascular disease burden in the diabetes patients has increased throughout the past four decades.[3] A cross-sectional study showed that HTN was commonly in newly diagnosed diabetes and HTN patients had a higher prevalence of cardiovascular events than normotensive subjects before the diagnosis of diabetes.[13] Hypertension has also been reported to be one of the strongest risk factors for cardiovascular diseases including coronary disease, vascular heart diseases, and cerebral stroke.[14-20] The co-existence of diabetes and HTN demonstrated a dramatically increased risk of cardiovascular disease by two to four times compared to the adults without HTN or diabetes.[3] Thus, diabetes and HTN are thought to be the bad companions and blood pressure control is critical in the diabetes patients combined with HTN. Besides, a study demonstrated that HTN had stronger association with atherosclerotic cardiovascular

For peer review only - http://bmjopen.bmj.com/site/about/guidelines.xhtml

disease than diabetes.[21]

Effect of hypertension and diabetes on the risk of cardiovascular disease have been investigated in American, Finnish, Japanese and Iranian population.[21-24] However, there is little evidence from large-scale study to assess the joint effect of HTN and diabetes on the risk of cardiovascular events in China. The purpose of the study was to evaluate the impact of HTN and T2DM on the risk of cardiovascular disease and stroke in the Chinese adults using the SuValue database.

METHODS

Study Design

SuValue database is a big-data hospital information system (HIS) database, including >90 million patients from 161 hospitals across 18 provinces in China.[25] This was a retrospective, cohort study designed to evaluate the risk of cardiovascular diseases and stroke in patients with T2DM and/or HTN from 2004 to 2015 in China real-world setting. Patients were included if they met the following criteria: (1) aged≥18; (2) newly diagnosed T2DM and/or HTN; (3) had the baseline examination records before or within 3 months at the first diagnosis (details of baseline examination were described in the Baseline Parameters section); (4) EMRs could be found in one year later after the first diagnosis of T2DM and/or HTN. Patients were excluded as follows: sex information missing; had been diagnosed with stroke, myocardial infarction, coronary heart disease, heart failure, had received coronary artery bypass grafting or percutaneous coronary intervention before the first diagnosis of T2DM and/or HTN; with abnormal kidney function (normal range for serum creatinine: 54-106 μmoI/L for

For peer review only - http://bmjopen.bmj.com/site/about/guidelines.xhtml

men; 44-97 μmol/L for women). We also included non-T2DM and non-HTN patients who performed the baseline examination but not from obstetrics and gynecology department, cancer department, neurology department and cardiology department. Authorization for SuValue database was obtained when the database was setup, so both ethics consideration and written informed patient consent were not needed for this analysis. Besides, all patients' electronic medical records (EMRs) were deidentified and anonymized when the SuValue database was constructed.

Baseline Parameters

Sex, age, triglyceride, total cholesterol, high-density lipoprotein cholesterol (HDL-C), low-density lipoprotein cholesterol (LDL-C), serum creatinine, fasting blood glucose, serum insulin, proteinuria, medication (antidiabetics and antihypertensive medications) at baseline were captured from EMRs before or within 3 months at the first diagnosis of T2DM and/or HTN.

Outcomes

We examined two outcomes of interest: combined vascular event (VE) and stroke. Combined VE include stroke, myocardial infarction, coronary heart disease, heart failure and coronary bypass, percutaneous coronary intervention. The outcomes were defined as the first event or last record before December 31, 2019 in the event-free cases according to the diagnosis in the patients' EMRs.

Statistical Analyses

The categorical variables were described using frequency and percentage. The continuous variables were described using mean (SD) if normally distributed or median

For peer review only - http://bmjopen.bmj.com/site/about/guidelines.xhtml

interquartile range if not. Baseline characteristics were compared using ANOVA or Chi-square tests. Kaplan–Meier survival analysis of the incidence of combined VE and stroke by the presence of T2DM and/or HTN was performed. Cox proportional hazards model was used to assess the association between diseases and each outcome, which was adjusted for cardiovascular risk factors including gender, age, triglyceride, total cholesterol, HDL-C and LDL-C. Unadjusted, sex- and age-adjusted and cardiovascular risk factors–adjusted hazard ratios were obtained. A $P<0.05$ (two-sided) was considered as statistically significant. All statistical analyses were performed using SAS 9.4 (SAS Institute, Cary, NC, USA).

Public and Patient involvement

Patients and/or the public were not involved in the design, or conduct, or reporting, or dissemination plans of this research.

RESULTS

For the current study, 8,012 patients with T2DM only, 9,653 patients with HTN only and 3,592 patients with both T2DM and HTN and 10,561 patients without T2DM or HTN were selected from the SuValue database. The median follow-up duration was 4.1 (2.4, 4.9) years. General characteristics of the study population at baseline are presented in Table 1. Comparing baseline characteristics between the four groups revealed significant differences except for total cholesterol (p=0.3506) and serum insulin (p= 0.6502).

Kaplan–Meier analyses demonstrated increased combined VE and stroke risk in the HTN group (Fig. 1). In unadjusted models, compared with non-HTN and non-T2DM

For peer review only - http://bmjopen.bmj.com/site/about/guidelines.xhtml

patients, the HR of T2DM was 1.747 for combined VE [95% confidence interval (CI) 1.566-1.949, P<0.0001] and 2.077 for stroke (95% CI 1.755-2.459, P<0.0001). Further adjustment with the age and sex and then with cardiovascular risk factors attenuated the association of T2DM with combined VE and stroke risk (HR 1.332, 95% CI 1.134-1.565 and HR 1.584, 95% CI 1.246-2.014, respectively; P<0.001, Table 2).

Kaplan–Meier analyses demonstrated increased combined VE and stroke risk in the HTN group (Fig. 1). HTN only group had more than 6-fold increased risk of combined VE (95% CI 5.712-6.830) and more than 8-fold increased risk of stroke (95% CI 7.517-9.934) in unadjusted models (all Ps<0.0001). These associations were attenuated but still significant after adjustment for age and sex and then other major cardiovascular risk factors for both combined VE and stroke risk (HR 3.244, 95% CI 2.946-3.572 and HR 4.543, 95% CI 3.918-5.268, respectively; all Ps<0.0001, Table 2).

Kaplan–Meier analyses demonstrated increased combined VE and stroke risk in the HTN and T2DM group (Fig. 1). For combined VE, the HR of HTN and T2DM group was 4.93 (95% CI 4.93-5.474). HTN and T2DM group increased the risk of stroke to 5.990 (95% CI 5.102-7.032) in unadjusted models (all Ps<0.0001). These associations were attenuated but still significant after adjustment for age and sex and then other major cardiovascular risk factors for both combined VE risk and stroke risk (HR 3.002, 95% CI 2.577-3.497 and HR 4.151, 95% CI 3.346-5.149, respectively; all Ps<0.0001, Table 2).

Compared with HTN only group, unadjusted HR for combined VE risk was 0.789 and 0.693 for stroke risk in the both T2DM and HTN group (all Ps<0.0001). But after

For peer review only - http://bmjopen.bmj.com/site/about/guidelines.xhtml

adjustment with major cardiovascular risk factors, there was no significant reduced risk for both T2DM and HTN group compared with HTN only group (Table 3).

In unadjusted model, HTN and T2DM group and HTN only group were associated with the combined VE and stroke risk compared with T2DM only. After adjustment with age and sex and major cardiovascular risk factors, both HTN and T2DM group and HTN only group were still significantly associated with combined VE and stroke risk (Table 4).

DISCUSSION

In this present study, having HTN and/or T2DM was significantly associated with combined VE and stroke before and after adjustment for major cardiovascular risk factors compared with the non-T2DM and non-HTN group. The association of T2DM with the risk of both combined VE and stroke attenuated after adjusting for major cardiovascular risk factors. Besides, HTN only group was more significantly associated with combined VE and stroke risk than T2DM only group.

To our known, our study firstly investigated the combined effect of HTN and T2DM in large cohorts in real world setting in Chinese patients. There are several studies conducted in Finland, American and Japan investigating different impact of HTN and diabetes on cardiovascular disease incidence and mortality.[21-23 26] HTN and/or T2DM were associated with the increased risk of combined VE and stroke in this study. The HR for cardiovascular disease was about 2 in the T2DM only group, which was lower than 3 reported in Framinghanm cohort of 1952-74.[11] Both HTN and T2DM have been shown to be the strong risk factors for cardiovascular disease mortality.[3]

For peer review only - http://bmjopen.bmj.com/site/about/guidelines.xhtml

In unadjusted analyses and adjustment model for major cardiovascular risk factors, we observed that patients with both T2DM and HTN showed increased risk of combined VE and stroke compared with those only with T2DM. These results suggest that the HTN conferred an enhanced risk of cardiovascular disease. Similarly, co-existence of HTN and T2DM conferred increased risk of cardiovascular diseases incidence compared with T2DM only adults.[3 27]

After adjustment for major cardiovascular risk factors, patients with T2DM and HTN were not significantly increased with the combined VE and stroke risk compared with HTN only. In this study, we included patients with HTN and/or T2DM having medication records. Antihypertension treatment such as angiotensin converting enzyme inhibitor, calcium-channel blockers, or β blockers have been shown to reduce the risk of cardiovascular diseases according to several randomized trials and meta-analysis researches.[28 29] A meta-analysis study demonstrated that antihypertensive treatment decreased the risk of cardiovascular disease and stroke among patients with a history of cardiovascular disease without HTN.[30] Thus, combined T2DM and HTN did not increase the risk of combined VE and stroke compared with HTN, which may be due to the antihypertensive treatment in this population.

Besides, we also observed that HTN only group was more associated with combined VE and stroke risk than T2DM group. The results were similar with previous report that HTN alone was more related with all-cause and atherosclerotic cardiovascular disease than T2DM alone in community-dwelling older adults.[21] A study performed in Iranian older adults showed that T2DM alone increased the coronary heart disease

For peer review only - http://bmjopen.bmj.com/site/about/guidelines.xhtml

incidence, stroke incidence and the all-cause mortality compared with HTN alone .[24] Thus, a prospective study was necessary to be performed for further analysis.

The current study has several significant strengths. First, this study included large number of patients. Second, data information about cardiovascular risk factors were collected in this study. However, there were several limitations in this study. First, BMI and life style such as smoking and alcohol drinking were not accessed through EMRs. Second, mortality data could not be accessed through the HIS system in hospitals.

CONCLUSIONS

In summary, HTN and/or T2DM was strongly associated with the increased risk of combined VE and stroke independent of conventional cardiovascular risk factors. T2DM seems not the risk of combined VE and stroke in HTN patients after adjusting for major cardiovascular risk factors. HTN was more significantly associated with combined VE and stroke risk than T2DM. However, a prospective study investigating the impact of HTN and/or T2DM on combined VE and stroke risk is needed to be performed.

For peer review only - http://bmjopen.bmj.com/site/about/guidelines.xhtml

ACKNOWLEDGEMENTS

None

FUNDING

The authors received no specific funding for this work.

AUTHOR CONTRIBUTIONS

HSM and YL designed the study, analyzed the data, and wrote the first draft of the manuscript. HSM, YL, JL, and YD revised it critically for important intellectual content and approved the final version.

DATA AVAILABILITY

All data relevant to the study were obtained from SuValue database which belongs to a third party and is not publicly available.

COMPETING INTERESTS

None.

ETHICS APPROVAL

All analysis was performed based on SuValue database. Authorization for SuValue database was obtained when the database was setup, so both ethics consideration and written informed patient consent were not needed for this analysis. Besides, all patients' electronic medical records (EMRs) were deidentified and anonymized when the SuValue database was constructed.

REFERENCES

1 Society CD. Guidelines for the prevention and treatment of type 2 diabetes in China (2017 edition). *J Chin J Diabetes* 2018;10:4-67.

2 Li Y, Teng D, Shi X*, et al.* Prevalence of diabetes recorded in mainland China using 2018 diagnostic criteria from the American Diabetes Association: national cross sectional study. *BMJ (Clinical research ed)* 2020;369:m997.

For peer review only - http://bmjopen.bmj.com/site/about/guidelines.xhtml

3 Ferrannini E, Cushman WC. Diabetes and hypertension: the bad companions. *The Lancet* 2012;380(9841):601-10.

4 Sun D, Zhou T, Heianza Y*, et al.* Type 2 Diabetes and Hypertension. *Circ Res* 2019;124(6):930-37.

5 Rask-Madsen C, Li Q, Freund B*, et al.* Loss of insulin signaling in vascular endothelial cells accelerates atherosclerosis in apolipoprotein E null mice. *Cell metabolism* 2010;11(5):379-89.

6 Rask-Madsen C, Buonomo E, Li Q*, et al.* Hyperinsulinemia does not change atherosclerosis development in apolipoprotein E null mice. *Arteriosclerosis, thrombosis, and vascular biology* 2012;32(5):1124-31.

7 Sarwar N, Gao P, Seshasai SR*, et al.* Diabetes mellitus, fasting blood glucose concentration, and risk of vascular disease: a collaborative meta-analysis of 102 prospective studies. *Lancet (London, England)* 2010;375(9733):2215-22.

8 Huxley R, Barzi F, Woodward M. Excess risk of fatal coronary heart disease associated with diabetes in men and women: meta-analysis of 37 prospective cohort studies. *BMJ (Clinical research ed)* 2006;332(7533):73-8.

9 Schramm TK, Gislason GH, Køber L*, et al.* Diabetes patients requiring glucose-lowering therapy and nondiabetics with a prior myocardial infarction carry the same cardiovascular risk: a population study of 3.3 million people. *Circulation* 2008;117(15):1945-54.

10 Almdal T, Scharling H, Jensen JS*, et al.* The independent effect of type 2 diabetes mellitus on ischemic heart disease, stroke, and death: a population-based study of 13,000 men and women with 20 years of follow-up. *Archives of internal medicine* 2004;164(13):1422-6.

11 Fox CS. Cardiovascular disease risk factors, type 2 diabetes mellitus, and the Framingham Heart Study. *Trends in cardiovascular medicine* 2010;20(3):90-5.

12 Fox CS, Coady S, Sorlie PD*, et al.* Trends in cardiovascular complications of diabetes. *JAMA* 2004;292(20):2495-9.

13 Hypertension in Diabetes Study (HDS): I. Prevalence of hypertension in newly

For peer review only - http://bmjopen.bmj.com/site/about/guidelines.xhtml

presenting type 2 diabetic patients and the association with risk factors for cardiovascular and diabetic complications. *J Hypertens* 1993;11(3):309-17.

14 Lewington S, Clarke R, Qizilbash N*, et al.* Age-specific relevance of usual blood pressure to vascular mortality: a meta-analysis of individual data for one million adults in 61 prospective studies. *Lancet (London, England)* 2002;360(9349):1903-13.

15 Britton KA, Gaziano JM, Djoussé L. Normal systolic blood pressure and risk of heart failure in US male physicians. *European journal of heart failure* 2009;11(12):1129-34.

16 Lawes CM, Rodgers A, Bennett DA*, et al.* Blood pressure and cardiovascular disease in the Asia Pacific region. *J Hypertens* 2003;21(4):707-16.

17 Brown DW, Giles WH, Greenlund KJ. Blood pressure parameters and risk of fatal stroke, NHANES II mortality study. *American journal of hypertension* 2007;20(3):338-41.

18 Aronow WS, Fleg JL, Pepine CJ*, et al.* ACCF/AHA 2011 expert consensus document on hypertension in the elderly: a report of the American College of Cardiology Foundation Task Force on Clinical Expert Consensus Documents developed in collaboration with the American Academy of Neurology, American Geriatrics Society, American Society for Preventive Cardiology, American Society of Hypertension, American Society of Nephrology, Association of Black Cardiologists, and European Society of Hypertension. *Journal of the American Society of Hypertension : JASH* 2011;5(4):259-352.

19 Ikeda A, Iso H, Yamagishi K*, et al.* Blood pressure and the risk of stroke, cardiovascular disease, and all-cause mortality among Japanese: the JPHC Study. *American journal of hypertension* 2009;22(3):273-80.

20 Lotfaliany M, Akbarpour S, Mozafary A*, et al.* Hypertension phenotypes and incident cardiovascular disease and mortality events in a decade follow-up of a Middle East cohort. *J Hypertens* 2015;33(6):1153-61.

21 Oh JY, Allison MA, Barrett-Connor E. Different impacts of hypertension and diabetes mellitus on all-cause and cardiovascular mortality in community-dwelling

For peer review only - http://bmjopen.bmj.com/site/about/guidelines.xhtml

older adults: the Rancho Bernardo Study. *J Hypertens* 2017;35(1):55-62.

22 Hu G, Jousilahti P, Tuomilehto J. Joint effects of history of hypertension at baseline and type 2 diabetes at baseline and during follow-up on the risk of coronary heart disease. *Eur Heart J* 2007;28(24):3059-66.

23 Kokubo Y, Okamura T, Watanabe M*, et al.* The combined impact of blood pressure category and glucose abnormality on the incidence of cardiovascular diseases in a Japanese urban cohort: the Suita Study. *Hypertens Res* 2010;33(12):1238-43.

24 Zafari N, Asgari S, Lotfaliany M*, et al.* Impact Of Hypertension versus Diabetes on Cardiovascular and All-cause Mortality in Iranian Older Adults: Results of 14 Years of Follow-up. *Sci Rep* 2017;7(1):14220.

25 Wang C, Gao Y, Zhu L*, et al.* Treatment Patterns in Patients With Newly Diagnosed Type 2 Diabetes in China: A Retrospective, Longitudinal Database Study. *Clinical therapeutics* 2019;41(8):1440-52.

26 Hu G, Sarti C, Jousilahti P*, et al.* The impact of history of hypertension and type 2 diabetes at baseline on the incidence of stroke and stroke mortality. *Stroke* 2005;36(12):2538-43.

27 Chen G, McAlister FA, Walker RL*, et al.* Cardiovascular outcomes in framingham participants with diabetes: the importance of blood pressure. *Hypertension* 2011;57(5):891-7.

28 Patel A, MacMahon S, Chalmers J*, et al.* Effects of a fixed combination of perindopril and indapamide on macrovascular and microvascular outcomes in patients with type 2 diabetes mellitus (the ADVANCE trial): a randomised controlled trial. *Lancet (London, England)* 2007;370(9590):829-40.

29 Turnbull F, Neal B, Algert C*, et al.* Effects of different blood pressure-lowering regimens on major cardiovascular events in individuals with and without diabetes mellitus: results of prospectively designed overviews of randomized trials. *Archives of internal medicine* 2005;165(12):1410-9.

30 Thompson AM, Hu T, Eshelbrenner CL*, et al.* Antihypertensive Treatment and Secondary Prevention of Cardiovascular Disease Events Among Persons Without

For peer review only - http://bmjopen.bmj.com/site/about/guidelines.xhtml

Hypertension: A Meta-analysis. *JAMA* 2011;305(9):913-22.

For peer review only

For peer review only - http://bmjopen.bmj.com/site/about/guidelines.xhtml

Table 1 Baseline characteristics of included patients.

	HTN only	T2DM only	T2DM and HTN	Non-T2DM and non-HTN	P value
**Total**	9,653	8,012	3,592	10561	
**Sex**					
Male	4,419 (45.8%)	4,006 (50.0%)	1,634 (45.5%)	4889 (46.3%)	-
Female	5,234 (54.2%)	4,006 (50.0%)	1,958 (54.5%)	5672 (53.7%)	-
**Age**					
18-29 (n, %)	79 (0.8%)	204 (2.5%)	31 (0.9%)	1372 (13.0%)	-
30-39 (n, %)	602 (6.2%)	831 (10.4%)	139 (3.9%)	2439 (23.1%)	-
40-49 (n, %)	1,856 (19.2%)	2,323 (29.0%)	553 (15.4%)	3227 (30.6%)	-
50-59 (n, %)	1,856 (20.7%)	2,297 (28.7%)	927 (25.8%)	1954 (18.5%)	-
60-69 (n, %)	2,552 (26.4%)	1,704 (21.3%)	1,194 (33.2%)	1179 (11.2%)	-
70-79 (n, %)	1,715 (17.8%)	556 (6.9%)	573 (16.0%)	336 (3.2%)	-
≥80 (n, %)	852 (8.8%)	97 (1.2%)	175 (4.9%)	54 (0.5%)	-
**Triglyceride (mmol/L)**	1.86 (1.54)	2.35 (2.55)	2.29 (1.85)	1.71 (1.65)	<.0001
**Total cholesterol (mmol/L)**	5.31 (1.19)	5.32 (1.41)	5.29 (1.28)	5.28 (1.15)	0.3506
**HDL-C (mmol/L)**	1.41 (0.41)	1.25 (0.45)	1.26 (0.39)	1.40 (0.39)	<.0001
**LDL-C (mmol/L)**	3.17 (1.00)	3.07 (1.03)	3.02 (0.98)	3.03 (0.90)	<.0001
**Serum creatinine (μmol/L)**	81.11 (39.35)	70.57 (23.94)	84.26 (59.38)	76.78 (20.42)	<.0001
**HbA1c (%)**	5.58 (1.38)	9.41 (3.11)	8.41 (2.63)	5.73 (1.31)	<.0001
**Fasting blood-glucose (mmol/L)**	5.76 (1.28)	11.15 (5.28)	9.88 (5.14)	5.58 (1.13)	<.0001
**Serum insulin (pmol/liter)**	95.63 (60.05)	117.64 (288.36)	78.43 (57.89)	9.98 (3.21)	0.6502
**Proteinuria (positive)**	310 (3.2%)	145 (1.8%)	126 (3.5%)	143 (1.4%)	<.0001
**Diabetes complications**					
Diabetic nephropathy	-	450 (5.6%)	446 (12.4%)	-	-
Diabetic retinopathy	-	313 (3.9%)	159 (4.4%)	-	-

For peer review only - http://bmjopen.bmj.com/site/about/guidelines.xhtml

Diabetic neuropathy	-	462 (5.8%)	225 (6.3%)	-	-
Diabetic lower limb vascular disease	-	7 (0.09%)	15 (0.42%)	-	-
Diabetic foot	-	80 (1.0%)	61 (1.7%)	-	-

HTN: hypertension; T2DM: type 2 diabetes mellitus; HDL-C: high density lipoprotein cholesterol; LDL-C: low density lipoprotein cholesterol. For continuous variables, data were presented as mean (SD) and P values for four groups were calculated using ANOVA test. For categorical variables, data were presented as number (frequency) and P values for four groups were calculated using Chi-square test.

For peer review only

For peer review only - http://bmjopen.bmj.com/site/about/guidelines.xhtml

Table 2 Association of type 2 diabetes mellitus (T2DM) and/or hypertension (HTN) with combined vascular event and stroke compared with non-T2DM and non-HTN group.

	Combined vascular event (VE)		Stroke	
	HR (95% CI)	P value	HR (95% CI)	P value
**Unadjusted**				
Non-T2DM and non-HTN	1 (reference)		1 (reference)	
T2DM only	1.747 (1.566, 1.949)	<.0001	2.077 (1.755, 2.459)	<.0001
HTN only	6.246 (5.712, 6.830)	<.0001	8.642 (7.517, 9.934)	<.0001
T2DM and HTN	4.930 (4.93, 5.474)	<.0001	5.990 (5.102, 7.032)	<.0001
**Age- and sex-adjusted**				
Non-T2DM and non-HTN	1 (reference)		1 (reference)	
T2DM only	1.258 (1.127, 1.405)	<.0001	1.464 (1.235, 1.735)	<.0001
HTN only	3.344 (3.046, 3.670)	<.0001	4.409 (3.817, 5.093)	<.0001
T2DM and HTN	2.595 (2.329, 2.892)	<.0001	3.021 (2.561, 3.563)	<.0001
**Risk factors–adjusted**				
Non-T2DM and non-HTN	1 (reference)		1 (reference)	
T2DM only	1.332 (1.134, 1.565)	0.0005	1.584 (1.246, 2.014)	<.0001
HTN only	3.244 (2.946, 3.572)	<.0001	4.543 (3.918, 5.268)	<.0001
T2DM and HTN	3.002 (2.577, 3.497)	<.0001	4.151 (3.346, 5.149)	<.0001

CI, confidence interval; VE, combined vascular event; T2DM, type 2 diabetes mellitus; HR, hazard ratio; HTN, hypertension. Risk factors include age, sex, triglycerides, total cholesterol, high density lipoprotein cholesterol, low density lipoprotein cholesterol.

For peer review only

For peer review only - http://bmjopen.bmj.com/site/about/guidelines.xhtml

Table 3 Association of type 2 diabetes mellitus (T2DM) and hypertension (HTN) with combined vascular event and stroke compared with HTN only.

	Combined vascular event (VE)		Stroke	
	HR (95% CI)	P value	HR (95% CI)	P value
**Unadjusted**				
HTN only	1 (reference)		1 (reference)	
T2DM and HTN	0.789 (0.732, 0.851)	<.0001	0.693 (0.624, 0.769)	<.0001
**Age- and sex-adjusted**				
HTN only	1 (reference)		1 (reference)	
T2DM and HTN	0.776 (0.720, 0.836)	<.0001	0.685 (0.617, 0.761)	<.0001
**Risk factors–adjusted**				
HTN only	1 (reference)		1 (reference)	
T2DM and HTN	0.925 (0.814, 1.052)	0.2352	0.914 (0.771, 1.082)	0.2959

CI, confidence interval; VE, combined vascular event; T2DM, type 2 diabetes mellitus; HR, hazard ratio; HTN, hypertension. Risk factors include age, sex, triglycerides, total cholesterol, high density lipoprotein cholesterol, low density lipoprotein cholesterol.

For peer review only

Table 4 Association of type 2 diabetes mellitus (T2DM) and/or hypertension (HTN) with combined vascular event and stroke compared with T2DM only.

	Combined vascular event (VE)		Stroke	
	HR (95% CI)	P value	HR (95% CI)	P value
**Unadjusted**				
T2DM only	1 (reference)		1 (reference)	
HTN only	3.575 (3.296, 3.877)	<.0001	4.160 (3.701, 4.675)	<.0001
T2DM and HTN	2.821 (2.558, 3.112)	<.0001	2.883 (2.504, 3.321)	<.0001
**Age- and sex-adjusted**				
T2DM only	1 (reference)		1 (reference)	
HTN only	2.657 (2.447, 2.885)	<.0001	3.011 (2.676, 3.389)	<.0001
T2DM and HTN	2.062 (1.868, 2.276)	<.0001	2.063 (1.789, 2.379)	<.0001
**Risk factors–adjusted**				
T2DM only	1 (reference)		1 (reference)	
HTN only	2.435 (2.113, 2.805)	<.0001	2.868 (2.341, 3.513)	<.0001
T2DM and HTN	2.253 (1.876, 2.706)	<.0001	2.620 (2.031, 3.380)	<.0001

CI, confidence interval; VE, combined vascular event; T2DM, type 2 diabetes mellitus; HR, hazard ratio; HTN, hypertension. Risk factors include age, sex, triglycerides, total cholesterol, high density lipoprotein cholesterol, low density lipoprotein cholesterol.

For peer review only

For peer review only - http://bmjopen.bmj.com/site/about/guidelines.xhtml

Figure legends

Figure 1 Kaplan-Meier survival curve of combined vascular event (A) and stroke (B) among different groups. HTN: hypertension, T2DM: type 2 diabetes mellitus.

For peer review only

For peer review only - http://bmjopen.bmj.com/site/about/guidelines.xhtml

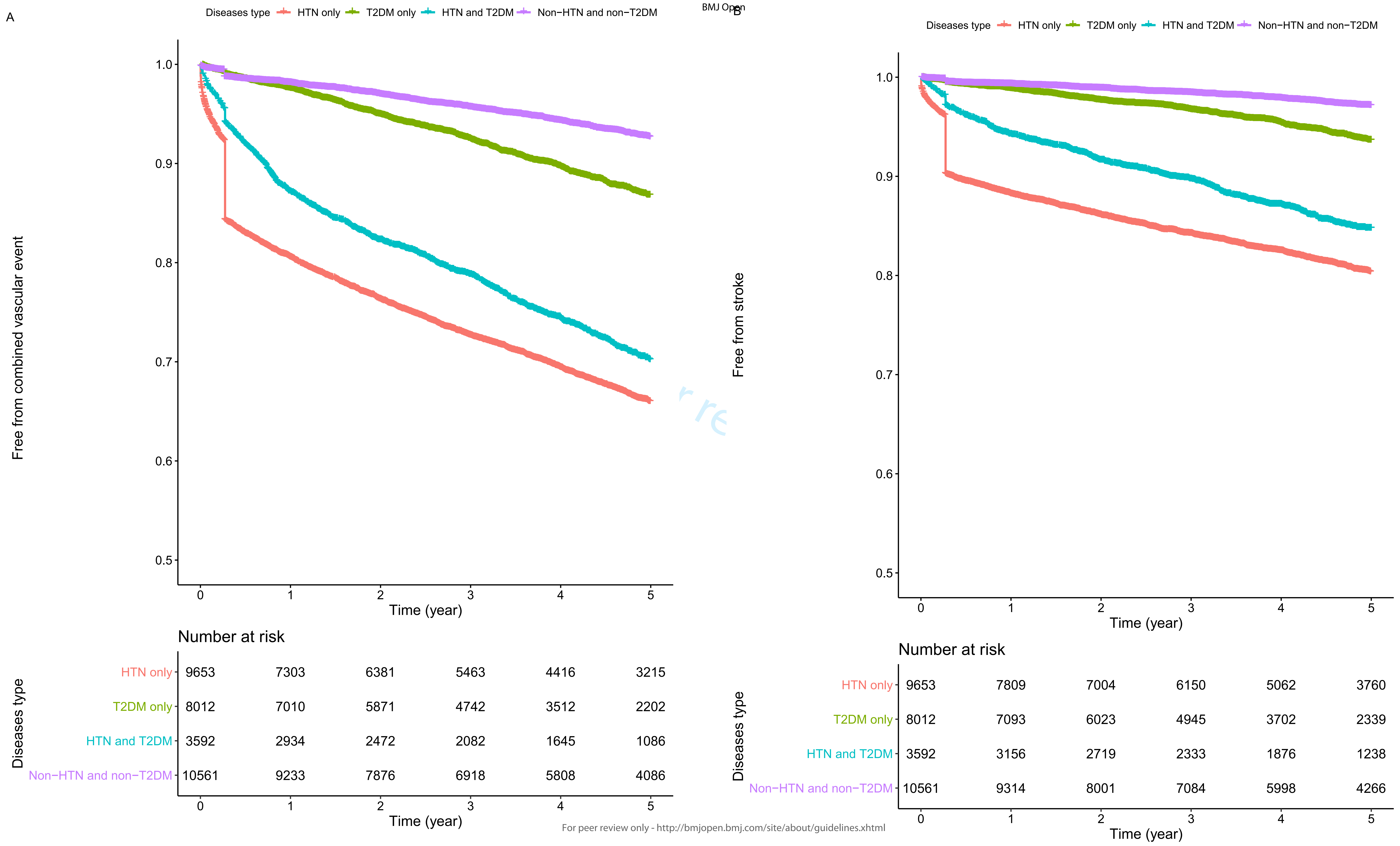
A
Diseases type
HTN only
T2DM only
HTN and T2DM
Non−HTN and non−T2DM
Free from combined vascular event
1.0
0.9
0.8
0.7
0.6
0.5
0
1
2
3
4
5
Time (year)
Number at risk
Diseases type
HTN only 9653 7303 6381 5463 4416 3215
T2DM only 8012 7010 5871 4742 3512 2202
HTN and T2DM 3592 2934 2472 2082 1645 1086
Non−HTN and non−T2DM 10561 9233 7876 6918 5808 4086
Time (year)
B
Diseases type
HTN only
T2DM only
HTN and T2DM
Non−HTN and non−T2DM
Free from stroke
1.0
0.9
0.8
0.7
0.6
0.5
0
1
2
3
4
5
Time (year)
Number at risk
Diseases type
HTN only 9653 7809 7004 6150 5062 3760
T2DM only 8012 7093 6023 4945 3702 2339
HTN and T2DM 3592 3156 2719 2333 1876 1238
Non−HTN and non−T2DM 10561 9314 8001 7084 5998 4266
Time (year)

STROBE Statement—Checklist of items that should be included in reports of ***cross-sectional studies***

	Item No	Recommendation	Page No
**Title and abstract**	1	(*a*) Indicate the study's design with a commonly used term in the title or the abstract	1, 2
		(*b*) Provide in the abstract an informative and balanced summary of what was done and what was found	2
**Introduction**			
Background/rationale	2	Explain the scientific background and rationale for the investigation being reported	4, 5
Objectives	3	State specific objectives, including any prespecified hypotheses	5
**Methods**			
Study design	4	Present key elements of study design early in the paper	5
Setting	5	Describe the setting, locations, and relevant dates, including periods of recruitment, exposure, follow-up, and data collection	5, 6
Participants	6	(*a*) Give the eligibility criteria, and the sources and methods of selection of participants	5, 6
Variables	7	Clearly define all outcomes, exposures, predictors, potential confounders, and effect modifiers. Give diagnostic criteria, if applicable	6
Data sources/ measurement	8*	For each variable of interest, give sources of data and details of methods of assessment (measurement). Describe comparability of assessment methods if there is more than one group	*5, 6*
Bias	9	Describe any efforts to address potential sources of bias	5, 6
Study size	10	Explain how the study size was arrived at	7
Quantitative variables	11	Explain how quantitative variables were handled in the analyses. If applicable, describe which groupings were chosen and why	5, 6
Statistical methods	12	(*a*) Describe all statistical methods, including those used to control for confounding	6, 7
		(*b*) Describe any methods used to examine subgroups and interactions	NA
		(*c*) Explain how missing data were addressed	NA
		(*d*) If applicable, describe analytical methods taking account of sampling strategy	NA
		(*e*) Describe any sensitivity analyses	NA
**Results**			
Participants	13*	(a) Report numbers of individuals at each stage of study—eg numbers potentially eligible, examined for eligibility, confirmed eligible, included in the study, completing follow-up, and analysed	7
		(b) Give reasons for non-participation at each stage	NA
		(c) Consider use of a flow diagram	NA
Descriptive data	14*	(a) Give characteristics of study participants (eg demographic, clinical, social) and information on exposures and potential confounders	7, 8
		(b) Indicate number of participants with missing data for each variable of interest	7, 8
Outcome data	15*	Report numbers of outcome events or summary measures	7, 8
Main results	16	(*a*) Give unadjusted estimates and, if applicable, confounder-adjusted estimates and their precision (eg, 95% confidence interval). Make clear which confounders were adjusted for and why they were included	7, 8

For peer review only - http://bmjopen.bmj.com/site/about/guidelines.xhtml

		(*b*) Report category boundaries when continuous variables were categorized	7, 8
		(*c*) If relevant, consider translating estimates of relative risk into absolute risk for a meaningful time period	NA
Other analyses	17	Report other analyses done—eg analyses of subgroups and interactions, and sensitivity analyses	NA
**Discussion**			
Key results	18	Summarise key results with reference to study objectives	9, 10
Limitations	19	Discuss limitations of the study, taking into account sources of potential bias or imprecision. Discuss both direction and magnitude of any potential bias	10
Interpretation	20	Give a cautious overall interpretation of results considering objectives, limitations, multiplicity of analyses, results from similar studies, and other relevant evidence	9, 10
Generalisability	21	Discuss the generalisability (external validity) of the study results	9, 10
**Other information**			
Funding	22	Give the source of funding and the role of the funders for the present study and, if applicable, for the original study on which the present article is based	11

*Give information separately for exposed and unexposed groups.

Note: An Explanation and Elaboration article discusses each checklist item and gives methodological background and published examples of transparent reporting. The STROBE checklist is best used in conjunction with this article (freely available on the Web sites of PLoS Medicine at http://www.plosmedicine.org/, Annals of Internal Medicine at http://www.annals.org/, and Epidemiology at http://www.epidem.com/). Information on the STROBE Initiative is available at www.strobe-statement.org.

For peer review only

For peer review only - http://bmjopen.bmj.com/site/about/guidelines.xhtml

BMJ Open

Impacts of type 2 diabetes mellitus and hypertension on the incidence of cardiovascular diseases and stroke in China real-world setting: A retrospective cohort study

Journal:	*BMJ Open*
Manuscript ID	bmjopen-2021-053698.R2
Article Type:	Original research
Date Submitted by the Author:	05-Nov-2021
Complete List of Authors:	Liu, Yan; The Third People's Hospital of Datong, Department of Endocrinology<br>Li, Jie; The Third People's Hospital of Datong, Department of Endocrinology<br>Dou, Ying; Ashermed Pharmaceutical Technology Co., Ltd., Department of Medicine<br>Ma, Hongshan; The Third People's Hospital of Datong, Department of Cardiology
**Primary Subject Heading**:	Diabetes and endocrinology
Secondary Subject Heading:	Public health
Keywords:	Hypertension < CARDIOLOGY, Stroke < NEUROLOGY, DIABETES & ENDOCRINOLOGY

SCHOLARONE™
Manuscripts

I, the Submitting Author has the right to grant and does grant on behalf of all authors of the Work (as defined in the below author licence), an exclusive licence and/or a non-exclusive licence for contributions from authors who are: i) UK Crown employees; ii) where BMJ has agreed a CC-BY licence shall apply, and/or iii) in accordance with the terms applicable for US Federal Government officers or employees acting as part of their official duties; on a worldwide, perpetual, irrevocable, royalty-free basis to BMJ Publishing Group Ltd ("BMJ") its licensees and where the relevant Journal is co-owned by BMJ to the co-owners of the Journal, to publish the Work in this journal and any other BMJ products and to exploit all rights, as set out in our licence.

The Submitting Author accepts and understands that any supply made under these terms is made by BMJ to the Submitting Author unless you are acting as an employee on behalf of your employer or a postgraduate student of an affiliated institution which is paying any applicable article publishing charge ("APC") for Open Access articles. Where the Submitting Author wishes to make the Work available on an Open Access basis (and intends to pay the relevant APC), the terms of reuse of such Open Access shall be governed by a Creative Commons licence – details of these licences and which Creative Commons licence will apply to this Work are set out in our licence referred to above.

Other than as permitted in any relevant BMJ Author's Self Archiving Policies, I confirm this Work has not been accepted for publication elsewhere, is not being considered for publication elsewhere and does not duplicate material already published. I confirm all authors consent to publication of this Work and authorise the granting of this licence.

For peer review only

Impacts of type 2 diabetes mellitus and hypertension on the incidence of cardiovascular diseases and stroke in China real-world setting: A retrospective cohort study

Yan Liu[1], Jie Li[1], Ying Dou[2], Hongshan Ma[3,*]

[1]Department of Endocrinology, The Third People's Hospital of Datong, Datong 037008, China.

[2]Department of Medicine, Ashermed Pharmaceutical Technology Co., Ltd., Shanghai 200030, China.

[3]Department of Cardiology, The Third People's Hospital of Datong, Datong 037008, China.

*Corresponding author

Hongshan Ma, Department of Cardiology, The Third People's Hospital of Datong, Datong 037008, China. Tel: +86-13681971726, Email: mahongshannew@163.com.

Short title: Impacts of T2DM and HTN on combined VE and stroke risk

For peer review only

ABSTRACT

Objective The prevalence of type 2 diabetes mellitus (T2DM) and hypertension (HTN) has notably increased in recent years. However, there is little evidence from large-scale studies assessing the joint effect of T2DM and HTN on the risk of cardiovascular events in China. This study was performed to investigate the association of T2DM and HTN with the incidence of combined vascular events (VEs) and stroke in China.

Design A retrospective cohort study.

Setting Data were collected from the SuValue database which includes the electronic medical records of >90 million patients from 161 hospitals across 18 provinces in China.

Participants Patients aged ≥18 with a diagnosis of T2DM and/or HTN were included. Non-T2DM and non-HTN patients were included in this study as controls.

Outcomes Incidence of combined VEs and stroke during the study.

Results In the current study, 8,012 patients with T2DM, 9,653 patients with HTN, 3,592 patients with both T2DM and HTN, and 10,561 patients without T2DM or HTN were included. T2DM was significantly associated with combined VE and stroke risk (hazard ratio 1.332, 95% CI 1.134-1.565 and HR 1.584, 95% CI 1.246-2.014, respectively). HTN was significantly associated with combined VE and stroke risk (HR 3.244, 95% CI 2.946-3.572 and HR 4.543, 95% CI 3.918-5.268, respectively). T2DM combined with HTN was significantly associated with combined VE and stroke risk (HR 3.002, 95% CI 2.577-3.497 and HR 4.151, 95% CI 3.346-5.149, respectively). HTN was associated with a higher combined VE and stroke risk than T2DM (HR 2.435, 95% CI 2.113-2.805 and HR 2.868, 95% CI 2.341-3.513, respectively).

For peer review only - http://bmjopen.bmj.com/site/about/guidelines.xhtml

Conclusion T2DM and HTN were strongly associated with combined VE and stroke risk; however, the HTN-only group had a higher combined VE and stroke risk than the T2DM-only group.

Keywords: type 2 diabetes mellitus, hypertension, cardiovascular events, stroke, vascular events

Strengths and limitations of this study

- This study was designed to analyse the electronic medical records of patients in a real-world setting.
- Cardiovascular risk factors were collected in this study.
- BMI and lifestyle factors, such as smoking and alcohol consumption, were not recorded in the electronic medical records of patients.
- Mortality data were not accessible through the electronic medical records of patients.

For peer review only

For peer review only - http://bmjopen.bmj.com/site/about/guidelines.xhtml

INTRODUCTION

The prevalence of type 2 diabetes mellitus (T2DM) increased to 10.4% in 2013 and 11.2% in 2015 from 0.67% in 1980 in China.[1 2] Hypertension (HTN) is found in over two-thirds of patients with diabetes.[3 4] The development of HTN coincides with the development of hyperglycaemia.[3] Insulin resistance and hyperinsulinaemia might promote atherogenesis, thereby affecting blood pressure homeostasis.[5 6]

Diabetes has been reported to be a strong risk factor for cardiovascular disease, all-cause mortality, coronary heart disease, ischemic heart disease and stroke in many studies.[7-11] According to the Framingham Heart Study, adults with diabetes had an absolute 2-fold increased risk of cardiovascular disease compared with subjects without diabetes.[12] The total cardiovascular disease burden in diabetes patients has increased throughout the past four decades.[3] A cross-sectional study showed that HTN was common in patients with newly diagnosed diabetes, and HTN patients had a higher prevalence of cardiovascular events than normotensive subjects before the diagnosis of diabetes.[13] Hypertension has also been reported to be one of the strongest risk factors for cardiovascular disease, including coronary disease, vascular heart disease, and cerebral stroke.[14-20] Adults with coexistent diabetes and HTN dramatically increased the risk of cardiovascular disease by two to four times compared to adults without HTN or diabetes.[3] Thus, diabetes and HTN are thought to be poor companions, and blood pressure control is critical in diabetes patients with HTN. In addition, a study demonstrated that HTN had a stronger association with atherosclerotic cardiovascular disease than diabetes.[21]

For peer review only

For peer review only - http://bmjopen.bmj.com/site/about/guidelines.xhtml

The effects of hypertension and diabetes on the risk of cardiovascular disease have been investigated in American, Finnish, Japanese and Iranian populations.[21-24] However, there is little evidence from large-scale studies assessing the joint effect of HTN and diabetes on the risk of cardiovascular events in China. The purpose of the study was to evaluate the impact of HTN and T2DM on the risk of cardiovascular disease and stroke in Chinese adults using the SuValue database.

METHODS

Study Design

The SuValue database is a big-data hospital information system (HIS) database that includes data on >90 million patients from 161 hospitals across 18 provinces in China.[25] This was a retrospective cohort study designed to evaluate the risk of cardiovascular diseases and stroke in patients with T2DM and/or HTN from 2004 to 2015 in a real-world Chinese setting. Patients were included if they met the following criteria: (1) aged ≥18; (2) newly diagnosed T2DM and/or HTN; (3) had baseline examination records before or within 3 months at the first diagnosis (details of baseline examination were described in the Baseline Parameters section); and (4) electronic medical records (EMRs) could be found one year later after the first diagnosis of T2DM and/or HTN. Patients were excluded as follows: missing sex information; had been diagnosed with stroke, myocardial infarction, coronary heart disease, heart failure; had received coronary artery bypass grafting or percutaneous coronary intervention before the first diagnosis of T2DM and/or HTN; and had abnormal kidney function (normal range for serum creatinine: 54-106 μmol/L for men; 44-97 μmol/L for women). We also included

For peer review only - http://bmjopen.bmj.com/site/about/guidelines.xhtml

non-T2DM and non-HTN patients who underwent the baseline examination but were not from the obstetrics and gynaecology department, cancer department, neurology department or cardiology department. Authorization for the SuValue database was obtained when the database was set up, so neither ethics review nor written informed patient consent were needed for this analysis. In addition, all patients' EMRs were deidentified and anonymized when the SuValue database was constructed.

Baseline Parameters

Sex, age, triglyceride, total cholesterol, high-density lipoprotein cholesterol (HDL-C), low-density lipoprotein cholesterol (LDL-C), serum creatinine, fasting blood glucose, serum insulin, proteinuria, and medication (antidiabetics and antihypertensive medications) at baseline were captured from EMRs before or within 3 months of the first diagnosis of T2DM and/or HTN.

Outcomes

We examined two outcomes of interest: combined vascular events (VEs) and stroke. Combined VEs include stroke, myocardial infarction, coronary heart disease, heart failure and coronary bypass, and percutaneous coronary intervention. The outcomes were defined as the first event or last record before December 31, 2019 among the event-free cases according to the diagnosis in the patients' EMRs.

Statistical Analyses

Categorical variables were described using frequencies and percentages. Continuous variables were described using the mean (SD) if normally distributed or as the median interquartile range if not. Baseline characteristics were compared using ANOVA or chi-

For peer review only - http://bmjopen.bmj.com/site/about/guidelines.xhtml

For peer review only

square tests. Kaplan–Meier survival analysis of the incidence of combined VEs and stroke according to the presence of T2DM and/or HTN was performed. A Cox proportional hazards model was used to assess the association between diseases and each outcome, which was adjusted for cardiovascular risk factors, including sex, age, triglycerides, total cholesterol, HDL-C and LDL-C. Unadjusted, sex- and age-adjusted and cardiovascular risk factor-adjusted hazard ratios were calculated. A $P<0.05$ (two-sided) was considered statistically significant. All statistical analyses were performed using SAS 9.4 (SAS Institute, Cary, NC, USA).

Public and Patient involvement

Neither patients nor the public were involved in the design, conduct, reporting, or dissemination plans of this research.

RESULTS

In the current study, 8,012 patients with T2DM-only, 9,653 patients with HTN-only and 3,592 patients with both T2DM and HTN and 10,561 patients without T2DM or HTN were selected from the SuValue database. The median follow-up duration was 4.1 (2.4, 4.9) years. The general characteristics of the study population at baseline are presented in Table 1. Comparing baseline characteristics between the four groups revealed significant differences, except in total cholesterol (P=0.3506) and serum insulin (p= 0.6502).

Kaplan–Meier analyses demonstrated increased combined VE and stroke risk in the HTN group (Fig. 1). In the unadjusted models, compared with non-HTN and non-T2DM, T2DM had an HR of 1.747 for combined VEs [95% confidence interval (CI)

For peer review only - http://bmjopen.bmj.com/site/about/guidelines.xhtml

1.566-1.949, P<0.0001] and 2.077 for stroke (95% CI 1.755-2.459, P<0.0001). Further adjustment with age and sex and then with cardiovascular risk factors attenuated the association of T2DM with combined VE and stroke risk (HR 1.332, 95% CI 1.134-1.565 and HR 1.584, 95% CI 1.246-2.014, respectively; P<0.001, Table 2).

Kaplan–Meier analyses demonstrated an increased combined VE and stroke risk in the HTN group (Fig. 1). The HTN-only group had a more than 6-fold increased risk of combined VE (95% CI 5.712-6.830) and a more than 8-fold increased risk of stroke (95% CI 7.517-9.934) in unadjusted models (all Ps<0.0001). These associations were attenuated but still significant after adjustment for age and sex and then for other major cardiovascular risk factors for both combined VE and stroke risk (HR 3.244, 95% CI 2.946-3.572 and HR 4.543, 95% CI 3.918-5.268, respectively; all Ps<0.0001, Table 2).

Kaplan–Meier analyses demonstrated increased combined VE and stroke risk in the HTN and T2DM groups (Fig. 1). For combined VEs, the HR of the HTN and T2DM group was 4.93 (95% CI 4.93-5.474). The HTN and T2DM groups had an increased risk of stroke to 5.990 (95% CI 5.102-7.032) in the unadjusted models (all Ps<0.0001). These associations were attenuated but still significant after adjustment for age and sex and then other major cardiovascular risk factors for both combined VE risk and stroke risk (HR 3.002, 95% CI 2.577-3.497 and HR 4.151, 95% CI 3.346-5.149, respectively; all Ps<0.0001, Table 2).

Compared with the HTN-only group, the unadjusted HRs for combined VE risk were 0.789 and 0.693 for stroke risk in both the T2DM and HTN groups (all Ps<0.0001). However, after adjustment for major cardiovascular risk factors, there was no

For peer review only - http://bmjopen.bmj.com/site/about/guidelines.xhtml

significantly reduced risk for either the T2DM or HTN group compared with the HTN-only group (Table 3).

In the unadjusted model, the HTN and T2DM group and the HTN-only group had a higher combined VE and stroke risk than the T2DM-only group. After adjustment for age and sex and major cardiovascular risk factors, both the HTN and T2DM groups and the HTN-only group were still significantly associated with combined VE and stroke risk (Table 4).

DISCUSSION

In the present study, having HTN and/or T2DM was significantly associated with combined VE and stroke before and after adjustment for major cardiovascular risk factors compared with the non-T2DM and non-HTN. The association of T2DM with the risk of both combined VE and stroke attenuated after adjusting for major cardiovascular risk factors. In addition, the HTN-only group had a higher combined VE and stroke risk than the T2DM-only group.

To our knowledge, our study is the first to investigate the combined effect of HTN and T2DM in a large cohort in a real-world setting in Chinese patients. Several studies investigating the different impacts of HTN and diabetes on cardiovascular disease incidence and mortality have been conducted in Finland, America and Japan.[21-23 26] HTN and/or T2DM were associated with an increased risk of combined VE and stroke in this study. The HR for cardiovascular disease was approximately 2 in the T2DM-only group, which was lower than the 3 reported in the Framingham cohort of 1952-74.[11] Both HTN and T2DM have been shown to be strong risk factors for cardiovascular

For peer review only - http://bmjopen.bmj.com/site/about/guidelines.xhtml

disease mortality.[3]

In unadjusted analyses and adjustment models for major cardiovascular risk factors, we observed that patients with both T2DM and HTN showed an increased risk of combined VE and stroke compared with those with T2DM alone. These results suggest that HTN confers an enhanced risk of cardiovascular disease. Similarly, the coexistence of HTN and T2DM conferred an increased risk of cardiovascular disease incidence compared with T2DM-only.[3 27]

After adjustment for major cardiovascular risk factors, patients with T2DM and HTN did not have a significantly increased combined VE and stroke risk compared with patients with HTN-only. In this study, we included patients with HTN and/or T2DM who had medication records. Antihypertension treatment, such as angiotensin converting enzyme inhibitors, calcium-channel blockers, or β blockers, has been shown to reduce the risk of cardiovascular diseases according to several randomized trials and meta-analysis studies.[28 29] A meta-analysis demonstrated that antihypertensive treatment decreased the risk of cardiovascular disease and stroke among patients with a history of cardiovascular disease without HTN.[30] Thus, combined T2DM and HTN did not increase the risk of combined VE and stroke compared with HTN, which may be due to the antihypertensive treatment in this population.

In addition, we also observed that the HTN-only group was more associated with combined VE and stroke risk than the T2DM group. The results were similar to a previous report that HTN alone was more related to all-cause and atherosclerotic cardiovascular disease than T2DM alone in community-dwelling older adults.[21] A

For peer review only - http://bmjopen.bmj.com/site/about/guidelines.xhtml

study performed in older Iranian adults showed that T2DM alone increased the coronary heart disease incidence, stroke incidence and all-cause mortality compared with HTN alone.[24] Thus, a prospective study is necessary for further analysis.

The current study has several significant strengths. First, this study included a large number of patients. Second, data about cardiovascular risk factors were collected in this study. However, there were several limitations in this study. First, BMI and lifestyle factors, such as smoking and alcohol consumption, were not recorded in the EMRs. Second, mortality data could not be accessed through the HIS system of hospitals.

CONCLUSIONS

In summary, HTN and/or T2DM was strongly associated with an increased risk of combined VE and stroke independent of conventional cardiovascular risk factors. T2DM does not seem to be a risk factor for combined VE and stroke in HTN patients after adjusting for major cardiovascular risk factors. HTN had a significantly higher combined VE and stroke risk than T2DM. However, a prospective study investigating the impact of HTN and/or T2DM on combined VE and stroke risk needs to be performed.

For peer review only - http://bmjopen.bmj.com/site/about/guidelines.xhtml

ACKNOWLEDGEMENTS

None

FUNDING

The authors received no specific funding for this work.

AUTHOR CONTRIBUTIONS

HSM and YL designed the study, analysed the data, and wrote the first draft of the manuscript. HSM, YL, JL, and YD revised it critically for important intellectual content and approved the final version.

DATA AVAILABILITY

All data relevant to the study were obtained from the SuValue database, which belongs to a third party and is not publicly available.

COMPETING INTERESTS

None.

ETHICS APPROVAL

All analyses were performed based on the SuValue database. Authorization for the SuValue database was obtained when the database was set up, so neither ethics review or written informed patient consent were needed for this analysis. In addition, all patients' electronic medical records (EMRs) were deidentified and anonymized when the SuValue database was constructed.

For peer review only

For peer review only - http://bmjopen.bmj.com/site/about/guidelines.xhtml

References

1 Society CD. Guidelines for the prevention and treatment of type 2 diabetes in China (2017 edition). *J Chin J Diabetes* 2018;10:4-67.

2 Li Y, Teng D, Shi X*, et al.* Prevalence of diabetes recorded in mainland China using 2018 diagnostic criteria from the American Diabetes Association: national cross sectional study. *BMJ (Clinical research ed)* 2020;369:m997.

3 Ferrannini E, Cushman WC. Diabetes and hypertension: the bad companions. *The Lancet* 2012;380(9841):601-10.

4 Sun D, Zhou T, Heianza Y*, et al.* Type 2 Diabetes and Hypertension. *Circ Res* 2019;124(6):930-37.

5 Rask-Madsen C, Li Q, Freund B*, et al.* Loss of insulin signaling in vascular endothelial cells accelerates atherosclerosis in apolipoprotein E null mice. *Cell metabolism* 2010;11(5):379-89.

6 Rask-Madsen C, Buonomo E, Li Q*, et al.* Hyperinsulinemia does not change atherosclerosis development in apolipoprotein E null mice. *Arteriosclerosis, thrombosis, and vascular biology* 2012;32(5):1124-31.

7 Sarwar N, Gao P, Seshasai SR*, et al.* Diabetes mellitus, fasting blood glucose concentration, and risk of vascular disease: a collaborative meta-analysis of 102 prospective studies. *Lancet (London, England)* 2010;375(9733):2215-22.

8 Huxley R, Barzi F, Woodward M. Excess risk of fatal coronary heart disease associated with diabetes in men and women: meta-analysis of 37 prospective cohort studies. *BMJ (Clinical research ed)* 2006;332(7533):73-8.

9 Schramm TK, Gislason GH, Køber L*, et al.* Diabetes patients requiring glucose-lowering therapy and nondiabetics with a prior myocardial infarction carry the same cardiovascular risk: a population study of 3.3 million people. *Circulation* 2008;117(15):1945-54.

10 Almdal T, Scharling H, Jensen JS*, et al.* The independent effect of type 2 diabetes mellitus on ischemic heart disease, stroke, and death: a population-based study of 13,000 men and women with 20 years of follow-up. *Archives of internal medicine*

For peer review only - http://bmjopen.bmj.com/site/about/guidelines.xhtml

2004;164(13):1422-6.

11 Fox CS. Cardiovascular disease risk factors, type 2 diabetes mellitus, and the Framingham Heart Study. *Trends in cardiovascular medicine* 2010;20(3):90-5.

12 Fox CS, Coady S, Sorlie PD*, et al.* Trends in cardiovascular complications of diabetes. *JAMA* 2004;292(20):2495-9.

13 Hypertension in Diabetes Study (HDS): I. Prevalence of hypertension in newly presenting type 2 diabetic patients and the association with risk factors for cardiovascular and diabetic complications. *J Hypertens* 1993;11(3):309-17.

14 Lewington S, Clarke R, Qizilbash N*, et al.* Age-specific relevance of usual blood pressure to vascular mortality: a meta-analysis of individual data for one million adults in 61 prospective studies. *Lancet (London, England)* 2002;360(9349):1903-13.

15 Britton KA, Gaziano JM, Djoussé L. Normal systolic blood pressure and risk of heart failure in US male physicians. *European journal of heart failure* 2009;11(12):1129-34.

16 Lawes CM, Rodgers A, Bennett DA*, et al.* Blood pressure and cardiovascular disease in the Asia Pacific region. *J Hypertens* 2003;21(4):707-16.

17 Brown DW, Giles WH, Greenlund KJ. Blood pressure parameters and risk of fatal stroke, NHANES II mortality study. *American journal of hypertension* 2007;20(3):338-41.

18 Aronow WS, Fleg JL, Pepine CJ*, et al.* ACCF/AHA 2011 expert consensus document on hypertension in the elderly: a report of the American College of Cardiology Foundation Task Force on Clinical Expert Consensus Documents developed in collaboration with the American Academy of Neurology, American Geriatrics Society, American Society for Preventive Cardiology, American Society of Hypertension, American Society of Nephrology, Association of Black Cardiologists, and European Society of Hypertension. *Journal of the American Society of Hypertension : JASH* 2011;5(4):259-352.

19 Ikeda A, Iso H, Yamagishi K*, et al.* Blood pressure and the risk of stroke, cardiovascular disease, and all-cause mortality among Japanese: the JPHC Study.

For peer review only - http://bmjopen.bmj.com/site/about/guidelines.xhtml

American journal of hypertension 2009;22(3):273-80.

20 Lotfaliany M, Akbarpour S, Mozafary A*, et al.* Hypertension phenotypes and incident cardiovascular disease and mortality events in a decade follow-up of a Middle East cohort. *J Hypertens* 2015;33(6):1153-61.

21 Oh JY, Allison MA, Barrett-Connor E. Different impacts of hypertension and diabetes mellitus on all-cause and cardiovascular mortality in community-dwelling older adults: the Rancho Bernardo Study. *J Hypertens* 2017;35(1):55-62.

22 Hu G, Jousilahti P, Tuomilehto J. Joint effects of history of hypertension at baseline and type 2 diabetes at baseline and during follow-up on the risk of coronary heart disease. *Eur Heart J* 2007;28(24):3059-66.

23 Kokubo Y, Okamura T, Watanabe M*, et al.* The combined impact of blood pressure category and glucose abnormality on the incidence of cardiovascular diseases in a Japanese urban cohort: the Suita Study. *Hypertens Res* 2010;33(12):1238-43.

24 Zafari N, Asgari S, Lotfaliany M*, et al.* Impact Of Hypertension versus Diabetes on Cardiovascular and All-cause Mortality in Iranian Older Adults: Results of 14 Years of Follow-up. *Sci Rep* 2017;7(1):14220.

25 Wang C, Gao Y, Zhu L*, et al.* Treatment Patterns in Patients With Newly Diagnosed Type 2 Diabetes in China: A Retrospective, Longitudinal Database Study. *Clinical therapeutics* 2019;41(8):1440-52.

26 Hu G, Sarti C, Jousilahti P*, et al.* The impact of history of hypertension and type 2 diabetes at baseline on the incidence of stroke and stroke mortality. *Stroke* 2005;36(12):2538-43.

27 Chen G, McAlister FA, Walker RL*, et al.* Cardiovascular outcomes in framingham participants with diabetes: the importance of blood pressure. *Hypertension* 2011;57(5):891-7.

28 Patel A, MacMahon S, Chalmers J*, et al.* Effects of a fixed combination of perindopril and indapamide on macrovascular and microvascular outcomes in patients with type 2 diabetes mellitus (the ADVANCE trial): a randomised controlled trial. *Lancet (London, England)* 2007;370(9590):829-40.

For peer review only - http://bmjopen.bmj.com/site/about/guidelines.xhtml

29 Turnbull F, Neal B, Algert C*, et al.* Effects of different blood pressure-lowering regimens on major cardiovascular events in individuals with and without diabetes mellitus: results of prospectively designed overviews of randomized trials. *Archives of internal medicine* 2005;165(12):1410-9.

30 Thompson AM, Hu T, Eshelbrenner CL*, et al.* Antihypertensive Treatment and Secondary Prevention of Cardiovascular Disease Events Among Persons Without Hypertension: A Meta-analysis. *JAMA* 2011;305(9):913-22.

For peer review only

For peer review only - http://bmjopen.bmj.com/site/about/guidelines.xhtml

Table 1 Baseline characteristics of the included patients.

	HTN-only	T2DM-only	T2DM and HTN	Non-T2DM and non-HTN	P value
**Total**	9,653	8,012	3,592	10561	
**Sex**					
Male	4,419 (45.8%)	4,006 (50.0%)	1,634 (45.5%)	4889 (46.3%)	-
Female	5,234 (54.2%)	4,006 (50.0%)	1,958 (54.5%)	5672 (53.7%)	-
**Age**					
18-29 (n, %)	79 (0.8%)	204 (2.5%)	31 (0.9%)	1372 (13.0%)	-
30-39 (n, %)	602 (6.2%)	831 (10.4%)	139 (3.9%)	2439 (23.1%)	-
40-49 (n, %)	1,856 (19.2%)	2,323 (29.0%)	553 (15.4%)	3227 (30.6%)	-
50-59 (n, %)	1,856 (20.7%)	2,297 (28.7%)	927 (25.8%)	1954 (18.5%)	-
60-69 (n, %)	2,552 (26.4%)	1,704 (21.3%)	1,194 (33.2%)	1179 (11.2%)	-
70-79 (n, %)	1,715 (17.8%)	556 (6.9%)	573 (16.0%)	336 (3.2%)	-
≥80 (n, %)	852 (8.8%)	97 (1.2%)	175 (4.9%)	54 (0.5%)	-
**Triglyceride (mmol/L)**	1.86 (1.54)	2.35 (2.55)	2.29 (1.85)	1.71 (1.65)	<.0001
**Total cholesterol (mmol/L)**	5.31 (1.19)	5.32 (1.41)	5.29 (1.28)	5.28 (1.15)	0.3506
**HDL-C (mmol/L)**	1.41 (0.41)	1.25 (0.45)	1.26 (0.39)	1.40 (0.39)	<.0001
**LDL-C (mmol/L)**	3.17 (1.00)	3.07 (1.03)	3.02 (0.98)	3.03 (0.90)	<.0001
**Serum creatinine (μmol/L)**	81.11 (39.35)	70.57 (23.94)	84.26 (59.38)	76.78 (20.42)	<.0001
**HbA1c (%)**	5.58 (1.38)	9.41 (3.11)	8.41 (2.63)	5.73 (1.31)	<.0001
**Fasting blood-glucose (mmol/L)**	5.76 (1.28)	11.15 (5.28)	9.88 (5.14)	5.58 (1.13)	<.0001
**Serum insulin (pmol/liter)**	95.63 (60.05)	117.64 (288.36)	78.43 (57.89)	9.98 (3.21)	0.6502
**Proteinuria (positive)**	310 (3.2%)	145 (1.8%)	126 (3.5%)	143 (1.4%)	<.0001
**Diabetes complications**					
Diabetic nephropathy	-	450 (5.6%)	446 (12.4%)	-	-
Diabetic retinopathy	-	313 (3.9%)	159 (4.4%)	-	-

For peer review only - http://bmjopen.bmj.com/site/about/guidelines.xhtml

Diabetic neuropathy	-	462 (5.8%)	225 (6.3%)	-	-
Diabetic lower limb vascular disease	-	7 (0.09%)	15 (0.42%)	-	-
Diabetic foot	-	80 (1.0%)	61 (1.7%)	-	-

HTN: hypertension; T2DM: type 2 diabetes mellitus; HDL-C: high density lipoprotein cholesterol; LDL-C: low density lipoprotein cholesterol. For continuous variables, data were presented as mean (SD) and P values for four groups were calculated using ANOVA test. For categorical variables, data were presented as number (frequency) and P values for four groups were calculated using Chi-square test.

For peer review only

For peer review only - http://bmjopen.bmj.com/site/about/guidelines.xhtml

Table 2 Association of type 2 diabetes mellitus (T2DM) and/or hypertension (HTN) with combined vascular events and stroke compared with the non-T2DM and non-HTN.

	Combined vascular event (VE)		Stroke	
	HR (95% CI)	P value	HR (95% CI)	P value
**Unadjusted**				
Non-T2DM and non-HTN	1 (reference)		1 (reference)	
T2DM-only	1.747 (1.566, 1.949)	<.0001	2.077 (1.755, 2.459)	<.0001
HTN-only	6.246 (5.712, 6.830)	<.0001	8.642 (7.517, 9.934)	<.0001
T2DM and HTN	4.930 (4.93, 5.474)	<.0001	5.990 (5.102, 7.032)	<.0001
**Age- and sex-adjusted**				
Non-T2DM and non-HTN	1 (reference)		1 (reference)	
T2DM-only	1.258 (1.127, 1.405)	<.0001	1.464 (1.235, 1.735)	<.0001
HTN-only	3.344 (3.046, 3.670)	<.0001	4.409 (3.817, 5.093)	<.0001
T2DM and HTN	2.595 (2.329, 2.892)	<.0001	3.021 (2.561, 3.563)	<.0001
**Risk factors–adjusted**				
Non-T2DM and non-HTN	1 (reference)		1 (reference)	
T2DM-only	1.332 (1.134, 1.565)	0.0005	1.584 (1.246, 2.014)	<.0001
HTN-only	3.244 (2.946, 3.572)	<.0001	4.543 (3.918, 5.268)	<.0001
T2DM and HTN	3.002 (2.577, 3.497)	<.0001	4.151 (3.346, 5.149)	<.0001

CI, confidence interval; VE, combined vascular event; T2DM, type 2 diabetes mellitus; HR, hazard ratio; HTN, hypertension. Risk factors include age, sex, triglycerides, total cholesterol, high density lipoprotein cholesterol, low density lipoprotein cholesterol.

For peer review only

For peer review only - http://bmjopen.bmj.com/site/about/guidelines.xhtml

Table 3 Association of type 2 diabetes mellitus (T2DM) and hypertension (HTN) with combined vascular events and stroke compared with HTN-only.

	**Combined vascular event (VE)**		**Stroke**	
	HR (95% CI)	P value	HR (95% CI)	P value
**Unadjusted**				
HTN-only	1 (reference)		1 (reference)	
T2DM and HTN	0.789 (0.732, 0.851)	<.0001	0.693 (0.624, 0.769)	<.0001
**Age- and sex-adjusted**				
HTN-only	1 (reference)		1 (reference)	
T2DM and HTN	0.776 (0.720, 0.836)	<.0001	0.685 (0.617, 0.761)	<.0001
**Risk factors–adjusted**				
HTN-only	1 (reference)		1 (reference)	
T2DM and HTN	0.925 (0.814, 1.052)	0.2352	0.914 (0.771, 1.082)	0.2959

CI, confidence interval; VE, combined vascular event; T2DM, type 2 diabetes mellitus; HR, hazard ratio; HTN, hypertension. Risk factors include age, sex, triglycerides, total cholesterol, high density lipoprotein cholesterol, low density lipoprotein cholesterol.

For peer review only

For peer review only - http://bmjopen.bmj.com/site/about/guidelines.xhtml

Table 4 Association of type 2 diabetes mellitus (T2DM) and/or hypertension (HTN) with combined vascular events and stroke compared with T2DM-only.

	Combined vascular event (VE)		Stroke	
	HR (95% CI)	P value	HR (95% CI)	P value
**Unadjusted**				
T2DM-only	1 (reference)		1 (reference)	
HTN-only	3.575 (3.296, 3.877)	<.0001	4.160 (3.701, 4.675)	<.0001
T2DM and HTN	2.821 (2.558, 3.112)	<.0001	2.883 (2.504, 3.321)	<.0001
**Age- and sex-adjusted**				
T2DM-only	1 (reference)		1 (reference)	
HTN-only	2.657 (2.447, 2.885)	<.0001	3.011 (2.676, 3.389)	<.0001
T2DM and HTN	2.062 (1.868, 2.276)	<.0001	2.063 (1.789, 2.379)	<.0001
**Risk factors–adjusted**				
T2DM-only	1 (reference)		1 (reference)	
HTN-only	2.435 (2.113, 2.805)	<.0001	2.868 (2.341, 3.513)	<.0001
T2DM and HTN	2.253 (1.876, 2.706)	<.0001	2.620 (2.031, 3.380)	<.0001

CI, confidence interval; VE, combined vascular event; T2DM, type 2 diabetes mellitus; HR, hazard ratio; HTN, hypertension. Risk factors include age, sex, triglycerides, total cholesterol, high density lipoprotein cholesterol, low density lipoprotein cholesterol.

For peer review only - http://bmjopen.bmj.com/site/about/guidelines.xhtml

Figure legends

Figure 1 Kaplan–Meier survival curve of combined vascular events (A) and stroke (B) among different groups. HTN: hypertension, T2DM: type 2 diabetes mellitus.

For peer review only

For peer review only - http://bmjopen.bmj.com/site/about/guidelines.xhtml

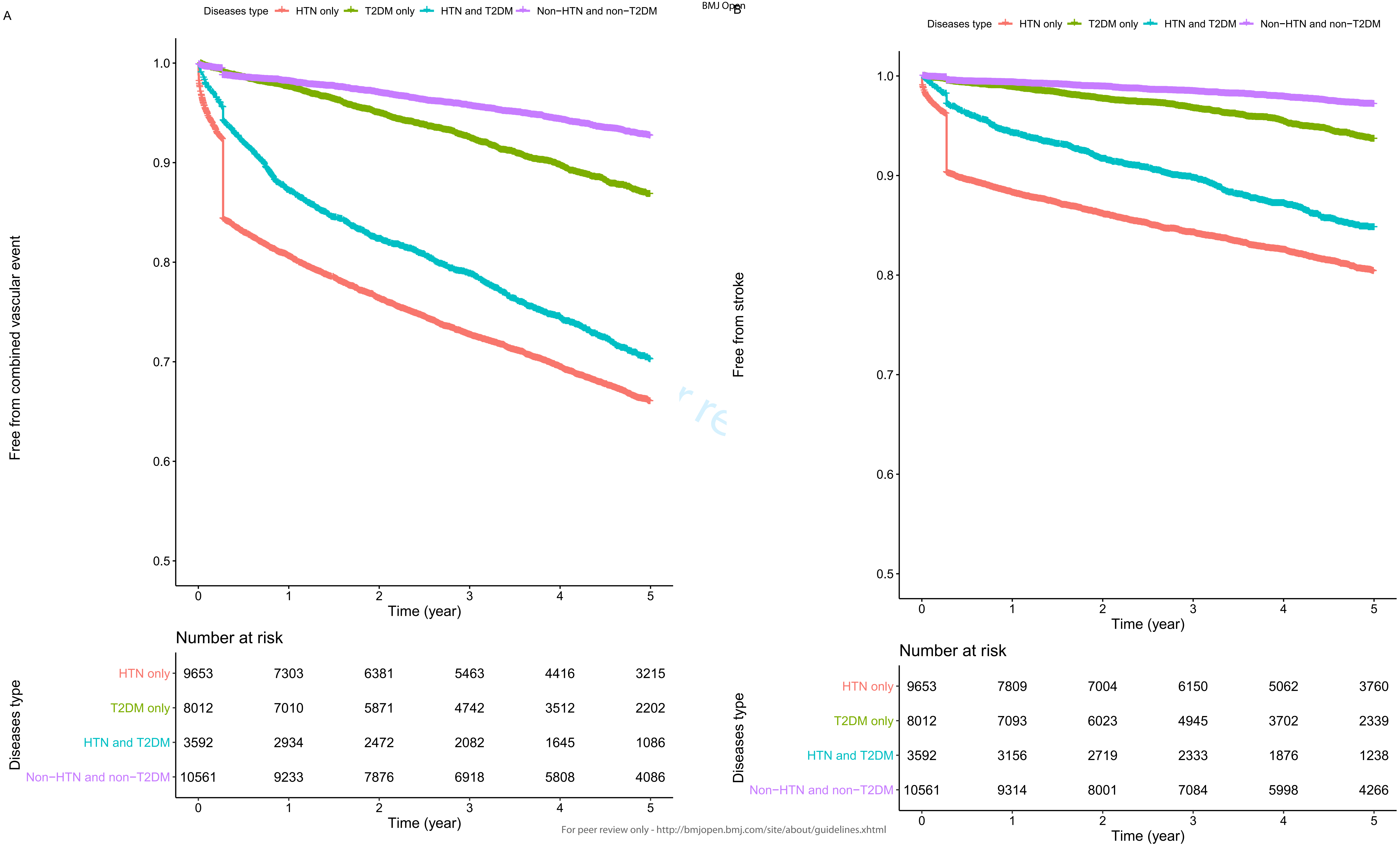
A
Diseases type HTN only T2DM only HTN and T2DM Non−HTN and non−T2DM
Free from combined vascular event
1.0
0.9
0.8
0.7
0.6
0.5
0 1 2 3 4 5
Time (year)
Number at risk
Diseases type
HTN only 9653 7303 6381 5463 4416 3215
T2DM only 8012 7010 5871 4742 3512 2202
HTN and T2DM 3592 2934 2472 2082 1645 1086
Non−HTN and non−T2DM 10561 9233 7876 6918 5808 4086
0 1 2 3 4 5
Time (year)
B
Diseases type HTN only T2DM only HTN and T2DM Non−HTN and non−T2DM
Free from stroke
1.0
0.9
0.8
0.7
0.6
0.5
0 1 2 3 4 5
Time (year)
Number at risk
Diseases type
HTN only 9653 7809 7004 6150 5062 3760
T2DM only 8012 7093 6023 4945 3702 2339
HTN and T2DM 3592 3156 2719 2333 1876 1238
Non−HTN and non−T2DM 10561 9314 8001 7084 5998 4266
0 1 2 3 4 5
Time (year)

STROBE Statement—Checklist of items that should be included in reports of ***cross-sectional studies***

	Item No	Recommendation	Page No
**Title and abstract**	1	(*a*) Indicate the study's design with a commonly used term in the title or the abstract	1, 2
		(*b*) Provide in the abstract an informative and balanced summary of what was done and what was found	2
**Introduction**			
Background/rationale	2	Explain the scientific background and rationale for the investigation being reported	4, 5
Objectives	3	State specific objectives, including any prespecified hypotheses	5
**Methods**			
Study design	4	Present key elements of study design early in the paper	5
Setting	5	Describe the setting, locations, and relevant dates, including periods of recruitment, exposure, follow-up, and data collection	5, 6
Participants	6	(*a*) Give the eligibility criteria, and the sources and methods of selection of participants	5, 6
Variables	7	Clearly define all outcomes, exposures, predictors, potential confounders, and effect modifiers. Give diagnostic criteria, if applicable	6
Data sources/ measurement	8*	For each variable of interest, give sources of data and details of methods of assessment (measurement). Describe comparability of assessment methods if there is more than one group	*5, 6*
Bias	9	Describe any efforts to address potential sources of bias	5, 6
Study size	10	Explain how the study size was arrived at	7
Quantitative variables	11	Explain how quantitative variables were handled in the analyses. If applicable, describe which groupings were chosen and why	5, 6
Statistical methods	12	(*a*) Describe all statistical methods, including those used to control for confounding	6, 7
		(*b*) Describe any methods used to examine subgroups and interactions	NA
		(*c*) Explain how missing data were addressed	NA
		(*d*) If applicable, describe analytical methods taking account of sampling strategy	NA
		(*e*) Describe any sensitivity analyses	NA
**Results**			
Participants	13*	(a) Report numbers of individuals at each stage of study—eg numbers potentially eligible, examined for eligibility, confirmed eligible, included in the study, completing follow-up, and analysed	7
		(b) Give reasons for non-participation at each stage	NA
		(c) Consider use of a flow diagram	NA
Descriptive data	14*	(a) Give characteristics of study participants (eg demographic, clinical, social) and information on exposures and potential confounders	7, 8
		(b) Indicate number of participants with missing data for each variable of interest	7, 8
Outcome data	15*	Report numbers of outcome events or summary measures	7, 8
Main results	16	(*a*) Give unadjusted estimates and, if applicable, confounder-adjusted estimates and their precision (eg, 95% confidence interval). Make clear which confounders were adjusted for and why they were included	7, 8

For peer review only

		(*b*) Report category boundaries when continuous variables were categorized	7, 8
		(*c*) If relevant, consider translating estimates of relative risk into absolute risk for a meaningful time period	NA
Other analyses	17	Report other analyses done—eg analyses of subgroups and interactions, and sensitivity analyses	NA
**Discussion**			
Key results	18	Summarise key results with reference to study objectives	9, 10
Limitations	19	Discuss limitations of the study, taking into account sources of potential bias or imprecision. Discuss both direction and magnitude of any potential bias	10
Interpretation	20	Give a cautious overall interpretation of results considering objectives, limitations, multiplicity of analyses, results from similar studies, and other relevant evidence	9, 10
Generalisability	21	Discuss the generalisability (external validity) of the study results	9, 10
**Other information**			
Funding	22	Give the source of funding and the role of the funders for the present study and, if applicable, for the original study on which the present article is based	11

*Give information separately for exposed and unexposed groups.

Note: An Explanation and Elaboration article discusses each checklist item and gives methodological background and published examples of transparent reporting. The STROBE checklist is best used in conjunction with this article (freely available on the Web sites of PLoS Medicine at http://www.plosmedicine.org/, Annals of Internal Medicine at http://www.annals.org/, and Epidemiology at http://www.epidem.com/). Information on the STROBE Initiative is available at www.strobe-statement.org.

For peer review only

For peer review only - http://bmjopen.bmj.com/site/about/guidelines.xhtml
